# Human papillomavirus infection and risk of cardiovascular disease: A population-based matched cohort study with sibling analysis

**DOI:** 10.1371/journal.pmed.1005222

**Published:** 2026-09-01

**Authors:** Yunyang Deng, Mark Clements, Chaonan Shen, Jonas F. Ludvigsson, Qing Shen, Jiayao Lei

**Affiliations:** 1 Department of Medical Epidemiology and Biostatistics, Karolinska Institutet, Stockholm, Sweden; 2 Clinical Research Unit, Shanghai Disabled Persons’ Federation Key Laboratory of Intelligent Rehabilitation Assistive Devices and Technologies, Shanghai Yangzhi Rehabilitation Hospital (Shanghai Sunshine Rehabilitation Center), Tongji University School of Medicine, Shanghai, China; 3 Laboratory of Mental Health Research, Tongji University School of Medicine, Shanghai, China; 4 Department of Paediatrics, Örebro University Hospital, Örebro, Sweden; 5 Department of Medicine, Columbia University College of Physicians and Surgeons, New York, New York, United States of America; 6 Institute for Advanced Study, Tongji University, Shanghai, China; 7 Unit of Integrative Epidemiology, Institute of Environmental Medicine, Karolinska Institutet, Stockholm, Sweden; 8 Department of Clinical Science, Intervention and Technology, Karolinska Institutet, Stockholm, Sweden; Peking University, CHINA

## Abstract

**Background:**

The role of human papillomavirus (HPV) in cardiovascular disease (CVD) remains unclear. We aimed to investigate the associations of HPV infection with incident CVD and CVD-related mortality.

**Methods and findings:**

In this population-based matched cohort study based on Swedish national registers, we identified women with an incident HPV infection during 2006 and 2024 (*N* = 497,445), and age-matched each woman with five women without infection. The time-varying associations between HPV infection and CVD were assessed using flexible parametric models, which also provided hazard ratios (HRs) and 95% confidence intervals (CIs) after adjusting for sociodemographic factors, HPV vaccination, chronic diseases, and parental history of related diseases. To control for familial factors, we also compared 143,787 women with HPV infection to their 174,637 unaffected full siblings.

A total of 28,793 and 128,373 incident CVDs were identified in women with and without HPV infection (Incidence: 8.27 and 7.93 per 1,000 person-years), respectively. Additionally, 1,221 and 4,941 CVD-related deaths were observed among women with and without HPV infection (Mortality: 33.73 and 29.39 per 100,000 person-years), respectively. The risks of incident CVD and mortality were most pronounced within the first follow-up year and attenuated thereafter.

Overall, women with HPV infection had an increased risk of incident CVD (adjusted HR 1.07, 95% CI [1.05, 1.08], absolute rate difference 0.53 per 1,000 person-years, population-attributable fraction 1.16%) and CVD-related mortality (adjusted HR 1.25, 95% CI [1.16, 1.34], absolute rate difference 6.71 per 100,000 person-years, population-attributable fraction 4.12%) than those without. During the first follow-up year, the adjusted HRs for incident CVD and CVD-related mortality were 1.43 (95% CI [1.38, 1.48]) and 1.86 (95% CI [1.41, 2.45]), respectively. Beyond the first follow-up year, the corresponding adjusted HRs decreased to 1.02 (95% CI [1.01, 1.04]) for incident CVD and 1.21 (95% CI [1.12, 1.30]) for CVD-related mortality. In sibling comparisons, positive associations with both outcomes were also observed. Women with HPV infection had fully adjusted HRs of 1.05 (95% CI [1.01, 1.08]) for incident CVD and 1.25 (95% CI [1.01, 1.53]) for CVD-related mortality compared to their siblings without HPV infections.

The main limitations of this study are its observational design, which precludes causal inference between HPV infection and CVD, and the potential misclassification of HPV infection, which may have biased the association towards the null.

**Conclusions:**

HPV infection was associated with increased risk of incident CVD and CVD-related mortality. The findings suggested that clinicians should be aware of a slightly elevated CVD risk in women with HPV, especially during the first year following an infection. However, given the small absolute difference, women should not be unduly concerned.

## Author summary

**Why was this study done?:**

- Human papillomavirus (HPV) infection is well known to cause cervical cancer, but emerging evidence suggests that it may also be associated with cardiovascular disease (CVD).
- Previous related observational studies were mainly based on selected healthcare populations and did not adequately account for shared familial factors.
- We conducted a nationwide matched cohort study in Sweden to examine whether women with a newly detected HPV infection were at increased risk of developing CVD or dying from CVD compared with women without infection.

**What did the researchers do and find?:**

- We followed 497,445 women with a newly detected HPV infection and 2,487,225 age-matched women without infection, and also compared 143,787 women with infection to their 174,637 full sisters without HPV infection.
- Women with HPV infection had a 7% higher risk of developing CVD and a 25% higher risk of dying from CVD than women without infection. The increase in risk was greatest during the first year after infection and was substantially smaller thereafter. However, the absolute risk increase was small.
- Similar associations were observed when comparing women with HPV infection to their sisters without the infection.

**What do these findings mean?:**

- Women with HPV infection may have a modestly increased risk of CVD, particularly during the first year after infection.
- Clinicians should be aware of the slightly increased short-term risk of CVD in women with HPV infection; however, given the small absolute increase in risk, women should not be unduly concerned.
- Because this was an observational study, it cannot prove that HPV infection directly causes CVD, and the potential misclassification of HPV infection may have biased the association towards the null.

## Abstract 3

In a large Swedish population study, Yunyang Deng and colleagues found that women with human papillomavirus (HPV) infection had slightly higher rates of cardiovascular disease and cardiovascular-related mortality than those without HPV, with the strongest associations observed during the first year after infection.

## Introduction

Cardiovascular diseases (CVDs) remain the primary cause of morbidity and mortality worldwide, with an estimated 17.9 million fatalities annually, contributing to ~32% of global deaths in 2019 [1]. Despite developments in identifying and managing traditional CVD risk factors, the disease continues to manifest in individuals without established risk factors [2]. Prior research has indicated that ~10%–20% of CVD cases occur in the absence of conventional contributors (e.g., diabetes, hyperlipidemia, and smoking, etc.), suggesting the need to explore additional risk factors [2].

Human papillomavirus (HPV) is the most common sexually transmitted virus, with an estimated lifetime infection rate exceeding 80% among sexually active individuals [3]. Globally, HPV infection is associated with ~4.5% occurrence of all cancers [4]. It is the primary cause of nearly all cervical cancers and significantly contributes to other anogenital and oropharyngeal malignancies [4]. Beyond its oncogenic potential, HPVs have been associated with the initiation of atherosclerotic diseases, either directly by infecting vascular cells and contributing to atherogenesis, or indirectly by infecting nonvascular cells and inducing systemic inflammation [5]. Recent observational studies from Korea [6,7] and the United States [8] have also indicated the positive associations of HPV infection with CVD incidence and/or mortality based on one or more healthcare centers. However, to the best of our knowledge, no population-based cohort study in Europe has been conducted. Moreover, no sibling-matched cohort study has been performed on this topic, despite its ability to control for unmeasured familial confounding, including shared genetic predispositions and early-life exposures.

Sweden presents a unique opportunity to investigate how HPV infections relate to incidence and mortality of CVD due to its well-established national health registers [9] and a well-organized national cervical cancer screening program with high participation rates (nearly 80% [10]) since the 1960s. The cervical screening program initially used cytology and later gradually transitioned to HPV-based screening from 2015 due to its higher sensitivity [11]. In this study, we aimed to examine the associations of HPV infection, defined as abnormal cervical screening results (abnormal cytology or positive HPV test), with incident CVD and CVD-related mortality in a nationwide population-based matched cohort and a sibling-matched cohort.

## Methods

### Data source

This study used nationwide Swedish population and healthcare registers linked at the individual level [12]. Prior to the study analyses, a prospective research plan was developed to guide the study design and analytical strategy and is provided as Supporting Information (S1 File). The changes made to the analyses during peer review, along with their corresponding rationales, have also been documented in the S1 File. Data on cervical cytology, HPV testing, and histology were retrieved from the Swedish National Cervical Screening Registry (NKCx), which has virtually complete nationwide coverage for all cervical screening results of women aged 23 (cervical screening starting age) or older since 1995 [11]. Diagnosis of CVD and other chronic diseases were retrieved from the Swedish National Patient Register, which offers nearly complete coverage of all inpatient care since 1987 and outpatient specialized care since 2001 [13]. Death information was collected from the Cause of Death Register, a high-quality, virtually complete national register of all deaths in Sweden since 1952 [14]. Cancer diagnoses were identified from the Swedish Cancer Register, a mandatory nationwide register established in 1958, which provides virtually complete coverage [15]. HPV vaccination data were collected from the Swedish HPV Vaccination Register, National Vaccination Register, and Prescribed Drug Register [16]. Information on parity and smoking during pregnancy were retrieved from the Medical Birth Registry, which was established in 1973 and included data on almost all births (gestational age ≥22 weeks) in Sweden [17]. Data on immigration, emigration, year of birth, country of birth, and county of residence were obtained from the Total Population Register [18]. Data regarding education and income were collected from the Longitudinal Integration Database for Health Insurance and Labor Market Studies [19]. We additionally conducted sibling comparisons, where women were linked to their family members via the Multi-Generation Register, which provides comprehensive familial linkage for individuals born since 1932 [20].

### Study population

**Fig 1:**
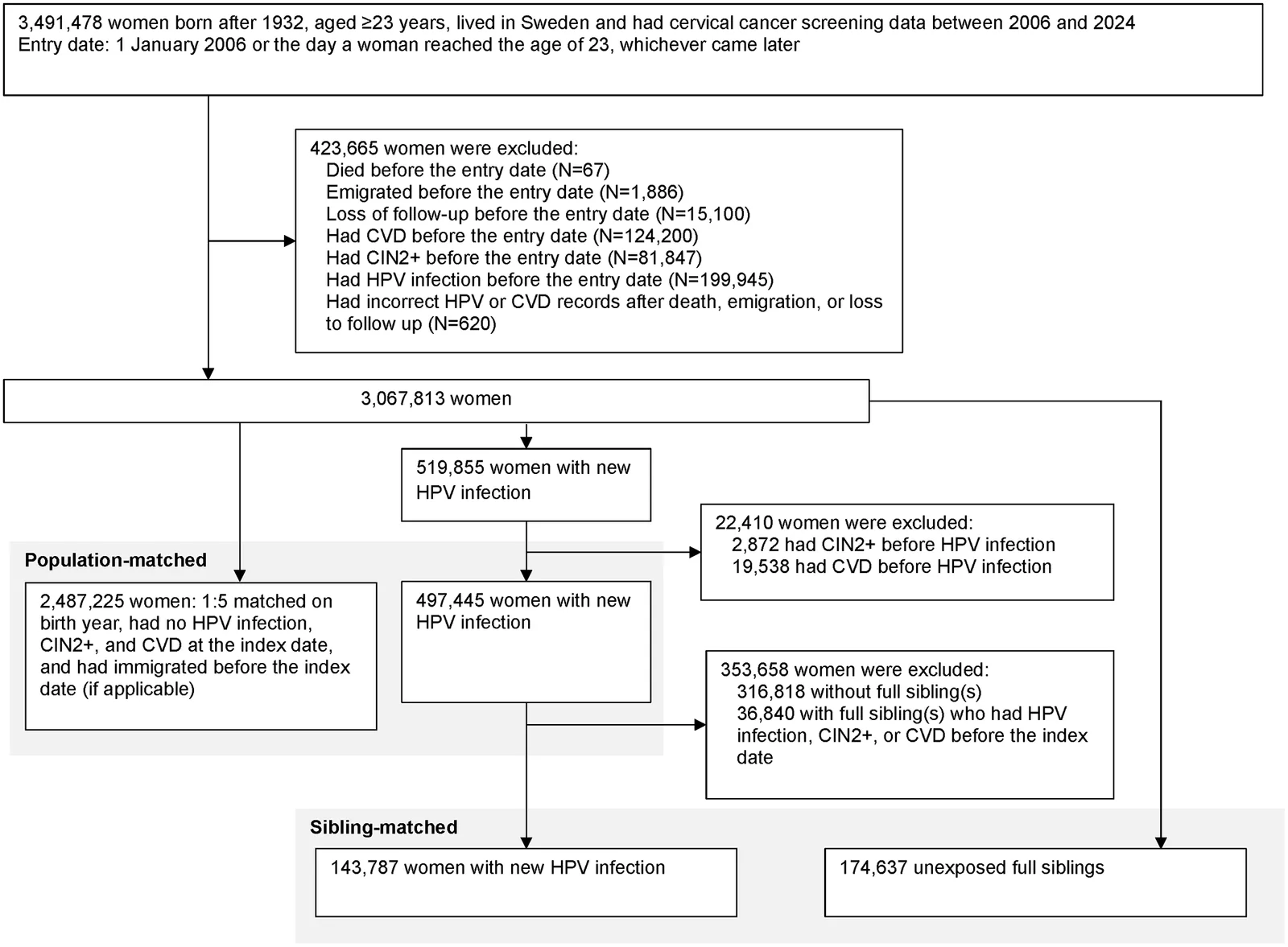
Flow chart of the study population. Abbreviations: CVD, cardiovascular disease; CIN, cervical intraepithelial neoplasia; HPV, human papillomavirus.

We identified 3,491,478 women born after 1932, aged ≥23 years (cervical screening starting age), and participated in cervical screening program (at least one cervical screening record for either cytology or HPV testing) between 2006 and 2024. The cohort entry was defined as January 1, 2006, or the date a woman turned 23 years old, whichever occurred later. We excluded 423,665 women with death, emigration, loss to follow-up, prior CVD diagnosis, cervical intraepithelial neoplasia grade 2 or higher (CIN2+), or HPV infection before the entry date, as well as those with incorrect HPV or CVD records after death, emigration, or loss to follow-up (Fig 1). Among the remaining 3,067,813 study population, those with a new HPV infection between 2006 and 2024 and without CIN2+ or CVD at the time of HPV infection were defined as the exposed group, with the date of HPV testing or cytology as the index date (*N* = 497,445).

In the population-matched cohort, each exposed woman was matched with five unexposed women, who were randomly selected from the study population-based on the year of birth (sampling with replacement). The unexposed women should be residents in Sweden, have no HPV infection, CIN2+, and CVD at the index date of the corresponding exposed woman (*N* = 2,487,225). Additionally, to further control for unmeasured familial confounding, we conducted sibling comparisons among exposed women with available full female siblings and compared the exposed women (*N* = 143,787) to their unaffected full siblings (*N* = 174,637) (Fig 1).

In both population-matched and sibling cohorts, each unexposed woman was assigned a pseudo-index date equal to the index date of the exposed woman to whom they were matched. Follow-up began on the index/pseudo-index date and continued until outcomes occurred, death, emigration, loss to follow-up, or end of study (31 December 2024), whichever came first. Unexposed women/unaffected siblings were further censored if having HPV infection during follow-up.

### HPV infection

In the NKCx, cytological results were classified as either normal or abnormal, with the latter defined as atypical squamous cells of undetermined significance or worse [21]. HPV testing results were categorized as either negative or positive for 14 high-risk HPV types (16, 18, 31, 33, 35, 39, 45, 51, 52, 56, 58, 59, 66, and 68). In this study, we defined women with either abnormal cytology or positive HPV testing results as exposed to HPV infection. To specific HPV infection and the index date, they were classified into four groups:

(1) Group 1: Women who had only cytology during follow-up. HPV infection was defined by the first abnormal cytological result, and its date of testing was used as the index date.
(2) Group 2: Women who had both cytology and HPV testing during follow-up with an interval less than 30 days in between. If HPV testing was positive, the woman was considered exposed regardless of cytology results, and the date of the first positive HPV testing was used as the index date. While if cytology was abnormal and HPV testing was negative, the woman was classified as not exposed. The 30-day threshold was used to minimize potential false positives in cytology.
(3) Group 3: Women who had both cytology and HPV testing during follow-up with a >30-day interval. The first abnormal result, either abnormal cytology or positive HPV testing, was used to define HPV infection, with its date as the index date.
(4) Group 4: Women who had only HPV testing during follow-up. HPV infection was defined by the first positive HPV testing, and its date was designated as the index date.

### Incident CVD and CVD-related mortality

For incident CVD, the primary outcome was the first primary or secondary diagnosis of any CVD. Incident CVD cases were first identified through the Swedish National Patient Register, and through the Cause of Death Register with CVD as the underlying cause of death if no proceeding CVD diagnosis existed, using International Classification of Diseases (ICD) codes. For CVD-related mortality, the primary outcome was the death with any CVD as the underlying cause. The CVDs were also grouped into subtypes including ischemic heart disease, cerebrovascular disease, embolism and thrombosis, hypertensive disease, heart failure, arrhythmia/conduction disorder, and other CVDs [22]. Moreover, major adverse cardiovascular event (MACE), defined as any of the following outcomes-incident ischemic heart disease, incident congestive heart failure, incident stroke, or cardiovascular death-was considered as an additional outcome [23]. All ICD codes are shown in S1 Table.

### Covariates

We included year of birth, country of birth, county of residence, the highest education level, annual household income level, high parity (>4), HPV vaccination, obesity, type 2 diabetes (T2D), hyperlipidemia, chronic obstructive pulmonary disease (COPD), cancers other than cervical cancer, mental disorders, maternal history of CIN3+, and parental history of CVD as covariates. These factors were selected because prior studies have reported their potential to confound the association between HPV and CVD (S1 Fig) [6–8,24]. For example, obesity, T2D, and hyperlipidemia were included for adjustment because previous studies showed that obesity and metabolic syndrome were positively associated with HPV infection and CVD, and that the associations between HPV infection and incident CVD/CVD-related mortality were stronger among women with obesity or metabolic syndrome than among those without [6,7]. Education was classified into three categories: low (less than high school), medium (high school), and high (university or above). Income was categorized as low, medium, and high, determined by the tertiles of income level among the population aged 20–65. All covariates except year of birth and country of birth were obtained from the year before the index/pseudo-index year. For any covariate with missing value, an additional category labeled “missing” was introduced. All relevant ICD codes are detailed in S1 Table.

### Statistical analysis

The characteristics for the whole cohort, exposed women with HPV infection (including the four exposure groups), and their matched controls, were presented as median with interquartile range (IQR) for continuous variables and as frequency (percentage) for categorical variables.

**Fig 2:**
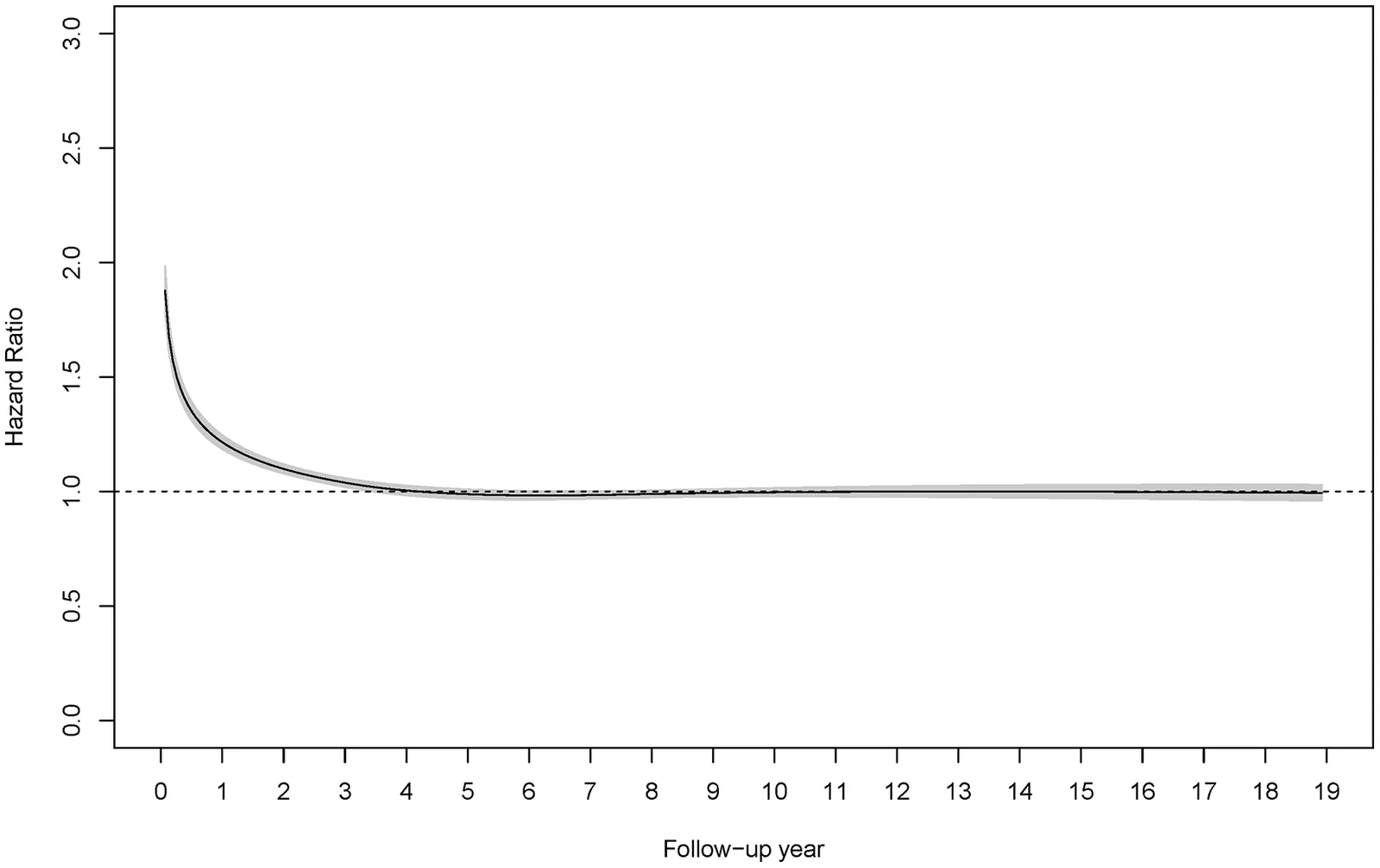
Time-varying HRs and 95% CIs for incident CVD associated with HPV infection in the population-matched cohort. Abbreviations: HR, hazard ratio; CI, confidence interval; CVD, cardiovascular disease; HPV, human papillomavirus.

**Fig 3:**
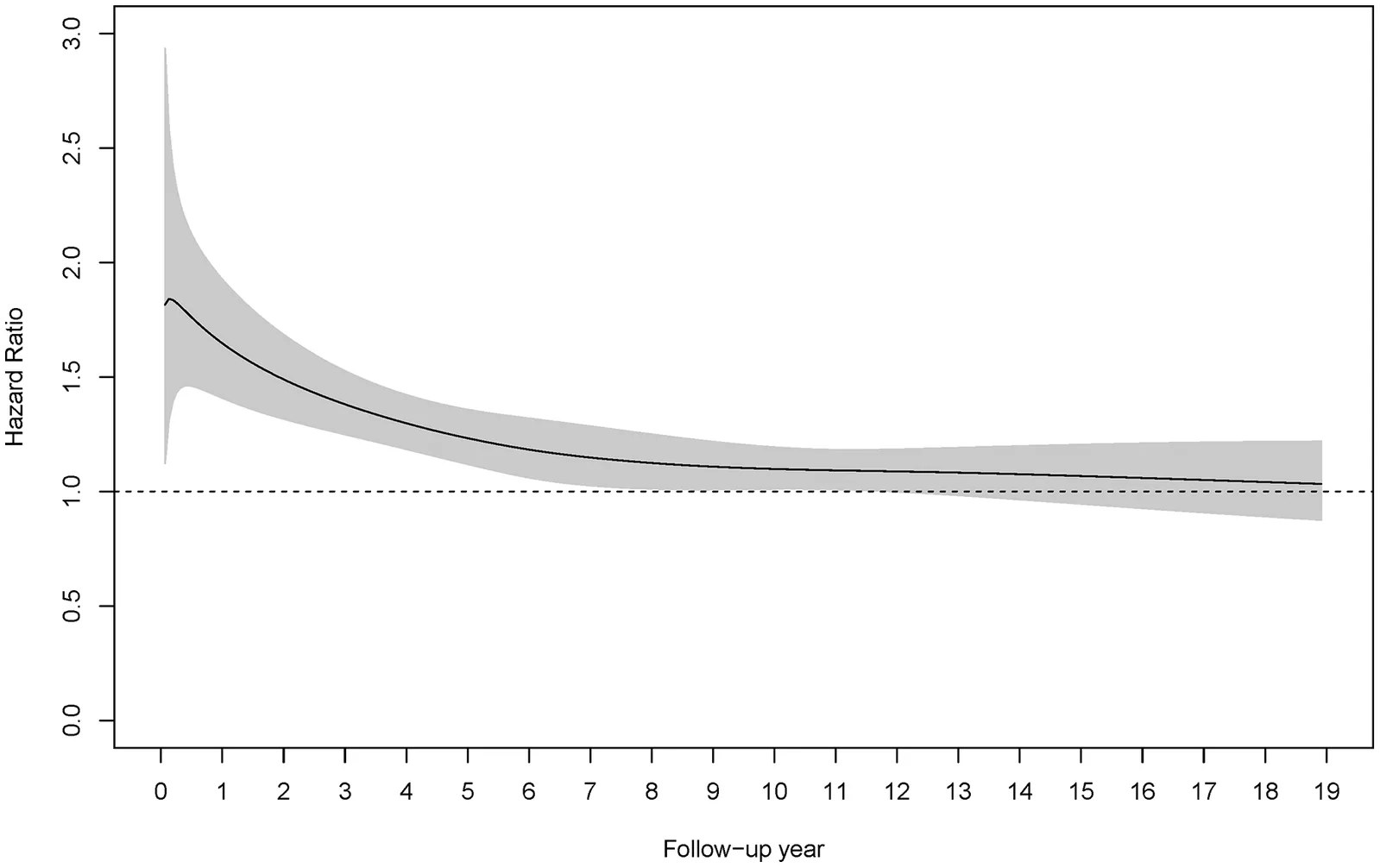
Time-varying HRs and 95% CIs for CVD-related mortality associated with HPV infection in the population-matched cohort. Abbreviations: HR, hazard ratio; CI, confidence interval; CVD, cardiovascular disease; HPV, human papillomavirus.

In the population-matched cohort, to visualize the temporal patterns for the associations of HPV infection with incident CVD and CVD-related mortality, we used flexible parametric models to generate time-varying associations. Since a substantially higher hazard ratio (HR) was observed during the first follow-up year compared with subsequent periods for both outcomes (Figs 2 and 3), we estimated the associations separately for the overall follow-up, <1 year, and ≥1 year. HRs and 95% confidence intervals (CIs) were calculated using flexible parametric models using time since the index/pseudo-index date as the underlying time scale. The flexible parametric survival models were specified on the log cumulative hazard scale with natural splines for log(time) (df = 4). For the population-matched cohort, we adjusted for year of birth using natural splines (df = 3) with robust variance estimation with individual-based clustering. Two models were generated: Model 1: crude model; Model 2: additionally adjusted for country of birth, county of residence, education, income, high parity, HPV vaccination, obesity, T2D, hyperlipidemia, COPD, cancers other than cervical cancer, mental disorders, maternal history of CIN3+, and parental history of CVD. Adjusted absolute rate differences (ARDs) and population-attributable fractions (PAFs) were estimated based on the fully adjusted HRs. The PAF was calculated as $p_{c}\times (\text{1}-\text{1}/HR)$, where $p_{c}$represents the proportion of exposed cases and HR denotes the adjusted HR. The variance of the PAF was calculated as $\left(\text{1}-\text{1}/HR{)}^{\text{2}}\times p_{c}\times (\text{1}-p_{c})/n_{c}+(p_{c}/HR{)}^{\text{2}}\times \text{var}(\text{log}HR\right)$, where $n_{c}\;$is the number of cases and var(logHR) represents the variance of the log HR.

We also conducted subgroup analyses based on age groups at the index date (<50 versus ≥ 50 years old), testing for exposure ascertainment (cytology or HPV testing), history of comorbidity (obesity, T2D, hyperlipidemia, COPD, cancers other than cervical cancer, or mental disorders) (yes or no), maternal history of CIN3+ (yes or no), parental history of CVD (yes or no), and HPV vaccination (yes or no). *P* values for interaction were obtained using Wald tests.

Additionally, we performed several sensitivity analyses: (1) Restricting analytic cohort to women who did not develop cervical cancer during follow-up to mitigate the potential increased risk of CVD due to cervical cancer treatment [6,7]. (2) Restricting outcomes to CVD cases identified only through primary diagnosis. (3) Restricting the analysis to women with parity ≥1, with an additional Model 3 that further adjusted for smoking during pregnancy, based on the covariates included in Model 2. This approach was adopted because smoking is a well-established risk factor for CVD [2], and smoking data are available only for women with a history of pregnancy from the Medical Birth Registry in our datasets [17]. (4) To address potential reverse causation, we conducted a sensitivity analysis excluding CVD events occurring within the first 3 months after the start of follow-up. (5) We defined HPV infection using two different cutoffs (15 and 60 days) for the intervals between cytology and HPV testing, to test the robustness of HPV infection definition.

In the sibling cohort, flexible parametric models were clustered by the sibling set shared between full siblings using a gamma frailty. HRs and 95% CIs were calculated based on the same covariates as in population-matched analyses plus year of birth, excluding maternal history of CIN3+ and parental history of CVD which were mutually controlled for in the sibling comparisons. Due to the limited sample size, analyses for CVD subtypes, subgroup analyses, and sensitivity analyses were not conducted in the sibling comparisons.

Data management was performed using SAS version 9.4. Statistical analyses were conducted using R version 4.4.1.

### Ethical statement

The study received ethical approval from the Stockholm Regional Ethics Review Board (Dnr. 02-556, 2011/921-32, 2025-01627-02), which waived written informed consent [25]. Adherence to the Strengthening the Reporting of Observational Studies in Epidemiology (STROBE) guidelines was maintained (S1 Checklist) [26].

### Disclosure of the use of artificial intelligence tools

No artificial intelligence tools were used to generate any content. ChatGPT 5.5 was used only for language improvement. The authors take full responsibility for the accuracy, integrity, and final content of the study.

## Results

### Population characteristics

In the population-matched cohort, a total of 2,984,670 women were included, comprising 497,445 exposed and 2,487,225 matched unexposed women. The median age at the index date was 31.78 (IQR [26.28, 42.37]) years old. The median follow-up duration was 5.52 years for incident CVD and 5.77 years for CVD-related mortality. Compared to their matched counterparts, women exposed to HPV infection were more likely to be born in Sweden, had lower annual household income, exhibited higher proportions of nulliparity, and were more likely to have a maternal history of CIN3+ (Table 1). Additionally, there were 143,787 exposed women and 174,637 unaffected siblings included in the sibling comparisons, with modest differences in the distribution of covariates (S2 Table). Furthermore, compared to the exposure Group 1, the other three groups (Groups 2–4) were younger at index date and had shorter follow-up time (S3 and S4 Tables).

**Table 1:** Study population characteristics in the population-matched cohort.

Variable^a^	Total	Matched group	Exposed group
Number	2,984,670	2,487,225	497,445
Median age at index date, years	31.78 [26.28, 42.37]	31.78 [26.28, 42.37]	31.78 [26.28, 42.36]
Median follow-up year for incident CVD	5.52 [2.63, 9.92]	5.34 [2.55, 9.80]	5.95 [2.91, 10.56]
Median follow-up year for CVD-related mortality	5.77 [2.75, 10.29]	5.67 [2.68, 10.20]	6.23 [3.09, 10.95]
Birth country			
Sweden	2,306,971 (77.29%)	1,907,379 (76.69%)	399,592 (80.33%)
Others	677,699 (22.71%)	579,846 (23.31%)	97,853 (19.67%)
Residence county			
Stockholm	716,616 (24.01%)	602,674 (24.23%)	113,942 (22.91%)
Others	2,244,870 (75.21%)	1,870,391 (75.20%)	374,479 (75.28%)
Missing	23,184 (0.78%)	14,160 (0.57%)	9,024 (1.81%)
Highest education level			
Low (less than high school)	251,801 (8.44%)	213,311 (8.58%)	38,490 (7.74%)
Medium (high school)	1,195,127 (40.04%)	990,431 (39.82%)	204,696 (41.15%)
High (university or above)	1,457,636 (48.84%)	1,220,728 (49.08%)	236,908 (47.62%)
Missing	80,106 (2.68%)	62,755 (2.52%)	17,351 (3.49%)
Annual household income level^b^			
Low	1,263,682 (42.34%)	1,021,566 (41.07%)	242,116 (48.67%)
Medium	927,321 (31.07%)	785,103 (31.57%)	142,218 (28.59%)
High	753,776 (25.25%)	652,974 (26.25%)	100,802 (20.26%)
Missing	39,891 (1.34%)	27,582 (1.11%)	12,309 (2.47%)
High parity (>4)			
No	1,375,020 (46.07%)	1,165,081 (46.84%)	209,939 (42.20%)
Yes	18,804 (0.63%)	15,679 (0.63%)	3,125 (0.63%)
Nulliparity	1,590,846 (53.30%)	1,306,465 (52.53%)	284,381 (57.17%)
Smoking during pregnancy			
No	1,049,261 (35.16%)	900,033 (36.19%)	149,228 (30.00%)
Yes	231,458 (7.75%)	185,566 (7.46%)	45,892 (9.23%)
Nulliparity	1,590,846 (53.30%)	1,306,465 (52.53%)	284,381 (57.17%)
Missing	113,105 (3.79%)	95,161 (3.83%)	17,944 (3.61%)
Obesity^c^	3,407 (0.11%)	2,788 (0.11%)	619 (0.12%)
Type 2 diabetes^c^	18,341 (0.61%)	15,517 (0.62%)	2,824 (0.57%)
Hyperlipidemia^c^	7,390 (0.25%)	6,148 (0.25%)	1,242 (0.25%)
Chronic obstructive pulmonary disease^c^	8,502 (0.28%)	6,859 (0.28%)	1,643 (0.33%)
Cancers other than cervical cancer^c^	67,852 (2.27%)	55,648 (2.24%)	12,204 (2.45%)
Mental disorders^c^	551,534 (18.48%)	449,050 (18.05%)	102,484 (20.60%)
Maternal history of CIN3 + ^c^	137,833 (4.62%)	109,075 (4.39%)	28,758 (5.78%)
Parental history of CVD^c^	897,665 (30.08%)	745,654 (29.98%)	152,011 (30.56%)
HPV vaccination^c^	483,313 (16.19%)	400,838 (16.12%)	82,475 (16.58%)

Abbreviations: CVD, cardiovascular disease; CIN, cervical intraepithelial neoplasia; HPV, human papillomavirus.

^a^The characteristics were reported as median [interquartile range] for continuous variables and as frequency (percentage) for categorical variables.

^b^Annual household income level was categorized into low, medium, and high based on the income level tertiles of the population aged 20–65.

^c^For binary variables with two levels (“yes” and “no”), only the “yes” category was displayed.

### Associations between HPV infection and incident CVD

In the population-matched cohort, 128,373 and 28,793 women with incident CVD were observed from the unexposed and exposed women, yielding the crude incidence rates (IRs) of 7.93 and 8.27 per 1,000 person-years (Table 2). We noted a peak in the risk increase of any incident CVD during the first follow-up year, and the risk was attenuated thereafter (Fig 2).

**Table 2:** Hazard ratios with 95% CIs for incident CVD associated with HPV infection.

Design and outcome	Number of events		Incidence [95% CI], per 1,000 person-years		Hazard ratio [95% CI]		aARD [95% CI]	aPAF [95% CI], %
	Matched group	Exposed group	Matched group	Exposed group	Model 1^a^	Model 2^b^		
**Population-matched cohort**								
**Total follow-up time**								
Any CVD	128,373	28,793	7.93 [7.88, 7.97]	8.27 [8.18, 8.37]	1.09 [1.08, 1.11]	1.07 [1.05, 1.08]	0.53 [0.42, 0.65]	1.16 [0.93, 1.38]
Ischemic heart disease	7,537	1,625	0.49 [0.48, 0.50]	0.49 [0.47, 0.51]	1.07 [1.02, 1.14]	1.03 [0.97, 1.09]	0.01 [−0.01, 0.04]	0.50 [−0.49, 1.50]
Cerebrovascular disease	9,265	2,122	0.60 [0.59, 0.61]	0.64 [0.61, 0.67]	1.13 [1.07, 1.18]	1.09 [1.03, 1.14]	0.05 [0.02, 0.09]	1.50 [0.64, 2.36]
Emboli and thrombosis	7,359	1,803	0.48 [0.47, 0.49]	0.54 [0.52, 0.57]	1.18 [1.12, 1.24]	1.13 [1.07, 1.19]	0.06 [0.03, 0.09]	2.23 [1.28, 3.19]
Hypertensive disease	60,916	13,361	3.86 [3.83, 3.89]	3.94 [3.88, 4.01]	1.08 [1.06, 1.10]	1.05 [1.03, 1.08]	0.21 [0.13, 0.29]	0.92 [0.59, 1.26]
Heart failure	2,870	632	0.19 [0.18, 0.19]	0.19 [0.18, 0.21]	1.08 [0.99, 1.18]	1.02 [0.93, 1.12]	0.00 [−0.01, 0.02]	0.36 [−1.29, 2.02]
Arrhythmia/conduction disorder	28,322	6,396	1.82 [1.80, 1.84]	1.91 [1.86, 1.96]	1.08 [1.05, 1.11]	1.07 [1.04, 1.10]	0.12 [0.07, 0.18]	1.17 [0.68, 1.66]
Other CVDs	12,104	2,854	0.78 [0.77, 0.80]	0.86 [0.83, 0.89]	1.13 [1.09, 1.18]	1.10 [1.06, 1.15]	0.08 [0.04, 0.12]	1.78 [1.04, 2.52]
**<1-year follow-up time**								
Any CVD	14,032	4,127	5.92 [5.82, 6.02]	8.35 [8.10, 8.61]	1.48 [1.43, 1.53]	1.43 [1.38, 1.48]	2.55 [2.25, 2.85]	6.83 [6.24, 7.43]
Ischemic heart disease	832	196	0.35 [0.33, 0.38]	0.40 [0.35, 0.46]	1.20 [1.03, 1.41]	1.12 [0.95, 1.32]	0.04 [−0.02, 0.11]	2.06 [−0.72, 4.85]
Cerebrovascular disease	1,019	230	0.43 [0.41, 0.46]	0.47 [0.41, 0.53]	1.14 [0.99, 1.32]	1.11 [0.96, 1.28]	0.05 [−0.02, 0.12]	1.79 [−0.66, 4.24]
Emboli and thrombosis	862	296	0.36 [0.34, 0.39]	0.60 [0.54, 0.67]	1.73 [1.51, 1.97]	1.65 [1.44, 1.89]	0.24 [0.15, 0.32]	10.06 [7.70, 12.43]
Hypertensive disease	6,073	2,185	2.57 [2.50, 2.63]	4.43 [4.25, 4.62]	1.83 [1.74, 1.92]	1.77 [1.68, 1.86]	1.97 [1.74, 2.20]	11.48 [10.62, 12.35]
Heart failure	295	94	0.12 [0.11, 0.14]	0.19 [0.16, 0.23]	1.60 [1.27, 2.02]	1.38 [1.07, 1.77]	0.05 [0.00, 0.09]	6.60 [2.00, 11.21]
Arrhythmia/conduction disorder	3,491	758	1.48 [1.43, 1.53]	1.54 [1.43, 1.65]	1.08 [1.00, 1.17]	1.06 [0.98, 1.15]	0.09 [−0.03, 0.22]	1.07 [−0.28, 2.41]
Other CVDs	1,460	368	0.62 [0.59, 0.65]	0.75 [0.67, 0.83]	1.26 [1.12, 1.41]	1.23 [1.09, 1.38]	0.14 [0.05, 0.23]	3.72 [1.77, 5.67]
**≥1-year follow-up time**								
Any CVD	114,341	24,666	8.27 [8.22, 8.32]	8.22 [8.12, 8.32]	1.05 [1.03, 1.06]	1.02 [1.01, 1.04]	0.18 [0.06, 0.30]	0.39 [0.14, 0.63]
Ischemic heart disease	6,705	1,429	0.51 [0.50, 0.52]	0.50 [0.47, 0.52]	1.06 [1.00, 1.13]	1.02 [0.96, 1.09]	0.01 [−0.02, 0.04]	0.35 [−0.71, 1.41]
Cerebrovascular disease	8,246	1,892	0.62 [0.61, 0.64]	0.66 [0.63, 0.69]	1.13 [1.07, 1.19]	1.09 [1.03, 1.15]	0.05 [0.02, 0.09]	1.50 [0.59, 2.41]
Emboli and thrombosis	6,497	1,507	0.49 [0.48, 0.50]	0.53 [0.50, 0.55]	1.11 [1.05, 1.18]	1.07 [1.01, 1.13]	0.03 [0.00, 0.06]	1.18 [0.13, 2.22]
Hypertensive disease	54,843	11,176	4.07 [4.03, 4.10]	3.82 [3.75, 3.89]	1.00 [0.98, 1.02]	0.97 [0.95, 1.00]	−0.10 [−0.19, −0.02]	−0.44 [−0.81, −0.06]
Heart failure	2,575	538	0.20 [0.19, 0.20]	0.19 [0.17, 0.20]	1.02 [0.93, 1.13]	0.98 [0.88, 1.08]	−0.00 [−0.02, 0.01]	−0.43 [−2.21, 1.36]
Arrhythmia/conduction disorder	24,831	5,638	1.87 [1.84, 1.89]	1.95 [1.90, 2.00]	1.08 [1.05, 1.12]	1.07 [1.04, 1.10]	0.13 [0.07, 0.19]	1.21 [0.69, 1.73]
Other CVDs	10,644	2,486	0.80 [0.79, 0.82]	0.87 [0.83, 0.90]	1.12 [1.07, 1.17]	1.09 [1.04, 1.14]	0.07 [0.03, 0.11]	1.50 [0.70, 2.30]
**Sibling-matched cohort**								
Any CVD	9,739	7,843	7.04 [6.91, 7.19]	7.06 [6.90, 7.21]	1.01 [0.98, 1.04]	1.05 [1.01, 1.08]	0.32 [0.07, 0.57]	1.92 [0.49, 3.36]

Abbreviations: CI, confidence interval; CVD, cardiovascular disease; HPV, human papillomavirus; aARD, adjusted absolute rate difference; aPAF, adjusted population-attributable fraction.

^a^Crude model.

^b^In the population-matched cohort, adjustments were made for the matching variable, birth country, residence county, education, annual family income, high parity, HPV vaccination, obesity, type 2 diabetes, hyperlipidemia, chronic obstructive pulmonary disease, cancers other than cervical cancer, mental disorders, maternal history of cervical intraepithelial neoplasia grade 3 or worse, and parental history of CVD. In the sibling-matched cohort, adjustments included the same covariates as in the population-matched cohort, with the addition of birth year, but excluded maternal history of cervical intraepithelial neoplasia grade 3 or worse and parental history of CVD.

**Fig 4:**
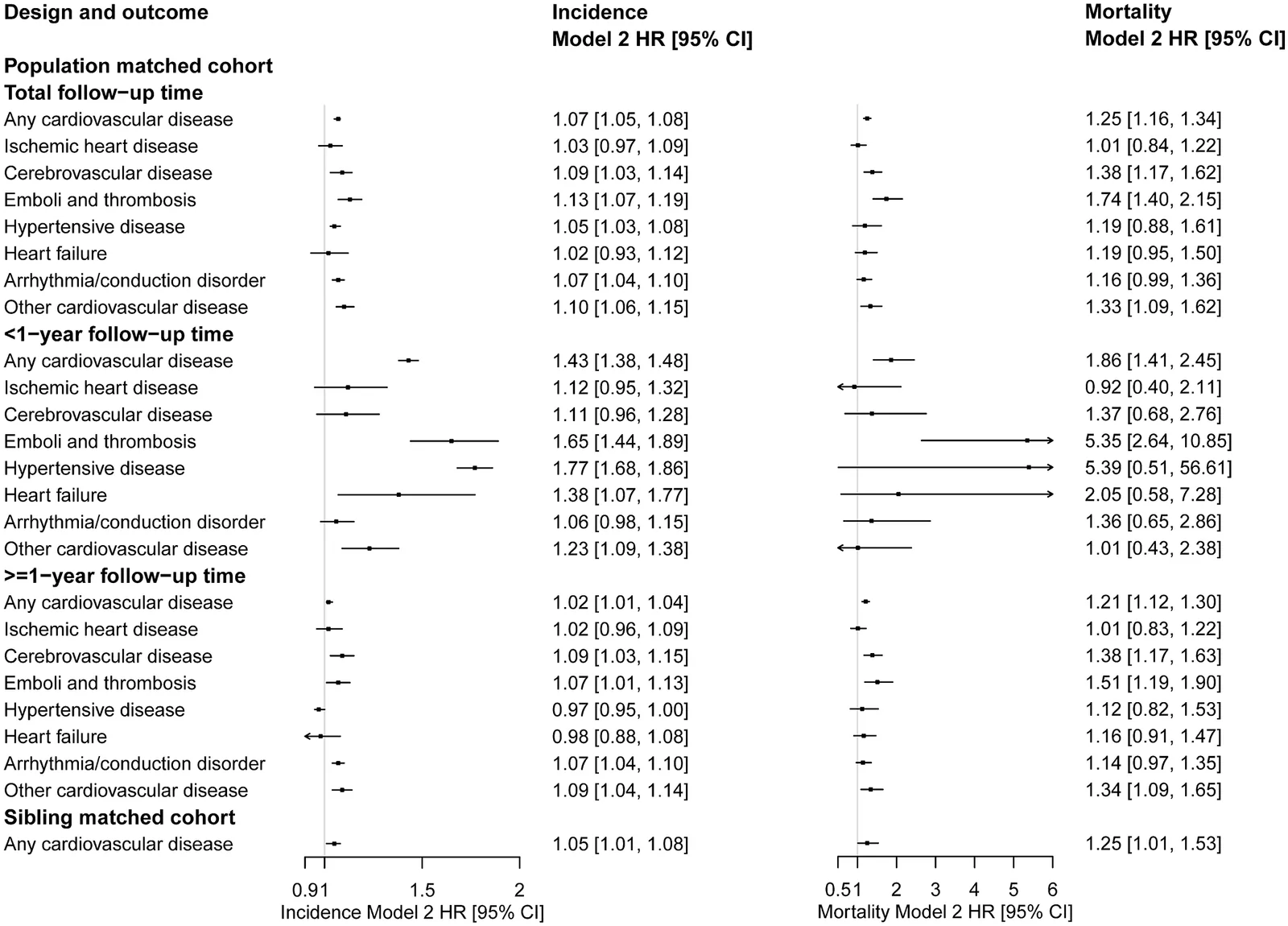
Fully adjusted HRs with 95% CIs for CVD associated with HPV infection. Abbreviations: HR, hazard ratio; CI, confidence interval; CVD, cardiovascular disease; HPV, human papillomavirus. In the population-matched cohort, adjustments were made for the matching variable, birth country, residence county, education, annual family income, high parity, HPV vaccination, obesity, type 2 diabetes, hyperlipidemia, chronic obstructive pulmonary disease, cancers other than cervical cancer, mental disorders, maternal history of cervical intraepithelial neoplasia grade 3 or worse, and parental history of CVD. In the sibling-matched cohort, adjustments included the same covariates as in the population-matched cohort, with the addition of birth year, but excluded maternal history of cervical intraepithelial neoplasia grade 3 or worse and parental history of CVD.

During the entire follow-up, the HRs of any CVD were 1.09 (95% CI [1.08, 1.11]) in Model 1 and 1.07 (95% CI [1.05, 1.08]) in Model 2 (Fig 4, Table 2). The adjusted ARD and PAF (Model 2) were 0.53 per 1,000 person-years (95% CI [0.42, 0.65]) and 1.16% (95% CI [0.93%, 1.38%]), respectively. When compared with unaffected full siblings, those exposed to HPV infection had a fully adjusted HR of 1.05 (95% CI [1.01, 1.08]) (Fig 4, Table 2). When examining CVD subtypes in the population-matched comparison, HPV infection was associated with an increased risk of cerebrovascular disease, embolism and thrombosis, hypertensive disease, arrhythmia/conduction disorder, and other CVDs (Fig 4, Table 2).

Moreover, in the population-based comparison, the fully adjusted HRs of any CVD were 1.43 (95% CI [1.38, 1.48]) during the first follow-up year and 1.02 (95% CI [1.01, 1.04]) beyond the first year of follow-up. For CVD subtypes, a significant association was found for embolism and thrombosis, hypertensive disease, heart failure, and other CVDs during the first follow-up year. After the first follow-up year, the risks of cerebrovascular disease, embolism and thrombosis, arrhythmia/conduction disorder, and other CVDs increased (Fig 4, Table 2).

In the subgroup analyses, the estimates of any CVD were more pronounced in women aged ≥50 years at index (vs. <50 years) or with parental CVD history (vs. without) (*P* for interaction <0.05). While no significant difference in the estimates for any CVD was observed when stratified by exposure testing method (cytology or HPV testing), history of comorbidity or mental disorders, maternal history of CIN3+, or HPV vaccination (S5 Table).

Sensitivity analyses showed that the association with any CVD remained unchanged when restricted to women without follow-up cervical cancer or when limited to women with primary CVD diagnoses compared to the main analyses. When the analysis was restricted to women with parity ≥1 and an additional Model 3 further adjusted for smoking during pregnancy based on the covariates in Model 2, the HRs (95% CIs) for any CVD were similar to those observed in the main analysis. Moreover, when excluding CVD events occurring within the first 3 months or using two different cutoffs (15 and 60 days) for the intervals between cytology and HPV testing to define HPV infection, the risk of any CVD was largely comparable to those in the main analysis (S6 Table).

### Associations between HPV infection and CVD-related mortality

In the population-matched cohort, 4,941 and 1,221 deaths due to CVD were found in the unexposed and exposed women, corresponding to crude mortality rates of 29.39 and 33.73 per 100,000 person-years (S7 Table). The HR for CVD-related mortality was highest during the first follow-up year, and the risk was attenuated thereafter (Fig 3).

Throughout the follow-up period, the HRs for any CVD were 1.34 (95% CI [1.25, 1.44]) in Model 1 and 1.25 (95% CI [1.16, 1.34]) in Model 2 (Fig 4, S7 Table). The adjusted ARD and PAF (Model 2) were 6.71 per 100,000 person-years (95% CI [4.24, 9.18]) and 4.12% (95% CI [2.88%, 5.35%]), respectively. We observed similar HRs for CVD-related mortality in the sibling cohort with a fully adjusted HR of 1.25 (95% CI [1.01, 1.53]) (Fig 4, S7 Table). For CVD-specific types, HPV infection was associated with an increased mortality of cerebrovascular disease, embolism and thrombosis, as well as other CVDs in the population-based comparison (Fig 4, S7 Table).

Additionally, in the population-based comparison, the fully adjusted HRs for any CVD were 1.86 (95% CI [1.41, 2.45]) within the first follow-up year and 1.21 (95% CI [1.12, 1.30]) beyond the first year of follow-up. During the first year of follow-up, increased mortality was observed for embolism and thrombosis. Beyond the first year, elevated mortality was noted for cerebrovascular disease, embolism and thrombosis, as well as other CVDs (Fig 4, S7 Table).

In the subgroup analyses, the association with any CVD-related mortality was stronger in women whose HPV infection was identified via cytology (vs. via HPV testing) (*P* for interaction <0.05). No significant difference in the estimates for any CVD-related mortality was observed when stratified by index age groups, history of comorbidity or mental disorders, maternal history of CIN3+ , parental history of CVD, or HPV vaccination (S8 Table).

Sensitivity analyses indicated that, when restricted to women without follow-up cervical cancer, the estimates of any CVD-related mortality were similar to those of main analyses. The estimates for any CVD-related mortality were higher when restricted to women with primary CVD diagnoses compared with main analyses. In contrast, when the analysis was limited to women with parity ≥1 and Model 3 was additionally adjusted for smoking during pregnancy, the HRs (95% CIs) for any CVD-related mortality were lower than those in main analyses but remained statistically significant. Moreover, when excluding CVD events occurring within the first 3 months or using two different cutoffs (15 and 60 days) for the intervals between cytology and HPV testing to define HPV infection, the estimates for any CVD were comparable to those observed in the main analysis (S9 Table).

### Associations between HPV infection and MACE

When assessing MACE, in the population-matched cohort, 23,195 events occurred among unexposed women and 5,246 among exposed women, corresponding to crude rates of 1.49 and 1.57 per 1,000 person-years, respectively. Over the follow-up, the HRs for MACE were 1.12 (95% CI [1.09, 1.16]) in Model 1 and 1.07 (95% CI [1.04, 1.11]) in Model 2. Fully adjusted HRs were 1.33 (95% CI [1.21, 1.45]) during the first follow-up year and 1.05 (95% CI [1.02, 1.09]) after the first year of follow-up (S10 Table).

## Discussion

In the population-based matched cohort, we found that HPV infection was associated with increased risk of both incident CVD and CVD-related mortality, with the risk peaking during the first year of follow-up and marginally increased thereafter. The increased risk of both incident CVD and CVD-related mortality remained significant in the sibling comparison. The findings suggested that clinicians should be aware of the slightly increased risk of CVD among women tested positive with HPV infection, especially during the early period (e.g., 1 year) following HPV infection. However, since the absolute difference was small, overall our findings are comforting, especially beyond the first year after HPV+.

Our findings are consistent with previous studies showing a positive association between HPV infection and risk of incident CVD and CVD-related mortality. A cohort study involving 163,250 CVD-free Korean women reported that those with high-risk HPV infection had higher CVD-related mortality compared to those without (adjusted HR = 1.68) [6]. Another cohort study of 63,411 Korean women found a significant association between high-risk HPV and incident CVD (adjusted HR = 1.25) [7]. Cross-sectional analyses of NHANES data also showed positive associations between vaginal HPV infection and CVD [8,24]. The lower HRs in our study than in the Korean studies may be due to differences in exposure definition. While the Korean studies relied on a single HPV test result, potentially capturing persistent infections acquired years earlier, we included only incident HPV infections during follow-up and excluded women with prior HPV infection or CVD. If persistent infections carry higher risk, this may explain the higher HRs in Korean studies. Although our study reported lower HRs compared to previous research, it further adds additional value by using a larger, population-based cohort with nationwide representation, allowing for more robust estimates.

Although increased risk was observed throughout the entire follow-up, our study demonstrated that the positive associations between HPV infection and incident CVD/CVD-related mortality were more evident during the first year of follow-up. Receiving an abnormal cervical screening result may lead to enhanced psychological stress, including anxiety and worrying towards uncertain health conditions, which could activate physiological stress responses such as hypothalamic-pituitary-adrenal axis dysregulation, increased cortisol secretion, and sympathetic nervous system activation [27]. These responses were associated with elevated blood pressure, endothelial dysfunction, and other processes implicated in CVD development [27]. This hypothesis was partially supported by the fact that a much higher proportion of incident mental disorders were diagnosed during the first 1–2 years of follow-up and decreased thereafter (S11 Table). Moreover, enhanced medical surveillance following an abnormal cervical screening result may facilitate earlier identification of certain CVD outcomes. Nonetheless, such increased monitoring is more likely to detect less severe CVD conditions, such as hypertension, emboli and thrombosis, and arrhythmia, while it is unlikely to account for the elevated risk observed for severe CVD types such as heart failure and cerebrovascular disease, or CVD mortality, or throughout the entire follow-up period. However, since we cannot rule out the possibility of surveillance bias, the elevated risk observed within the first year should be interpreted with caution.

Heterogeneity across CVD subtypes was observed in this study. One potential reason is that only the first CVD record during follow-up was captured for each participant, which may preferentially include less severe cases. Additionally, given that the study population is relatively young at baseline, diagnosed CVDs are more likely to be less severe, as more severe CVDs generally occur in middle-aged or older individuals [1]. Also, some CVD subtypes may be associated with mental disorders [28], which may act as a mediator in the association between HPV infection and CVD in our study.

The mechanisms linking HPV infection to CVD risk remain poorly understood. The viral oncoproteins E6 and E7 play a pivotal role by interfering with tumor suppressor pathways, including tumor protein p53 and retinoblastoma protein, thereby not only promoting cellular immortalization but also inducing endothelial dysfunction. This endothelial impairment, together with chronic low-grade inflammation triggered by HPV infection, fosters a pro-atherogenic environment. Furthermore, HPV-infected cells could secrete pro-inflammatory cytokines, including interleukin-1β (IL-1β), IL-6, and tumor necrosis factor-alpha (TNF-α), which further amplify vascular inflammation and facilitate atherosclerotic plaque development. Moreover, extracellular vesicles released from HPV-infected cells may contain viral DNA, oncoproteins, and microRNAs, transferring these molecules to endothelial and immune cells and thereby mediating horizontal gene transfer that exacerbates endothelial dysfunction and vascular inflammation. Oxidative stress represents an additional critical mechanism, as HPV infection elevates the production of reactive oxygen species, impairing vascular repair processes and aggravating endothelial injury. Additionally, HPV may alter lipid metabolism, resulting in dyslipidemia characterized by increased low-density lipoprotein (LDL) and very-low-density lipoprotein (VLDL) levels and decreased high-density lipoprotein (HDL) levels, thereby promoting cholesterol crystal accumulation and accelerating atherogenesis [29].

Our study has some strengths. First, the large sample size combined with the use of high-quality Swedish nationwide registries substantially minimizes the risk of selection and information biases, enhancing the validity and robustness of our findings [13]. Second, the near-complete coverage of cervical screening data enables accurate identification of HPV infections. This comprehensive data also allowed for the exclusion of women with a prior history of HPV infection, ensuring that all HPV cases represent new infections during follow-up and thereby facilitating a clear temporal sequence between HPV infection and subsequent CVD outcomes. Third, we adjusted for a wide range of covariates that are known to be associated with both HPV infection and CVD, thereby reducing the potential for residual confounding. Fourth, we analyzed the temporal associations between HPV infection and CVD risk over the follow-up period, which could identify the time window with the highest risk increase. Lastly, by employing a sibling comparison, we were able to partially control for confounding from factors shared within families, such as genetic background, early-life exposures, and correlated lifestyle behaviors, which may contribute to CVD risk and potentially influence both incident CVD and CVD-related mortality. The sibling-matched analysis therefore offered a more rigorous assessment of the associations between HPV infection and CVD. The similar estimates observed between the population-matched and sibling-matched analyses suggested that shared familial factors were unlikely to fully explain the associations between HPV infection and CVD observed in this study.

Nevertheless, this study has several limitations. First, since HPV testing data were only available from 2007 and recommended as the primary screening method starting in 2015 in Sweden [11], we utilized both cytology and HPV testing to ascertain HPV infection, which may have introduced misclassification bias. Specifically, the lower sensitivity of cytology may have led to nondifferential misclassification, potentially resulting in an underestimation of HPV infection and biasing the observed association with CVD towards null. In addition, the cutoff used to define HPV infection for the intervals between cytology and HPV testing was defined based on our understanding of an expected timeframe in screening setting as being ~30 days. Whereas in the sensitivity analyses, we defined HPV infection using two alternative cutoffs (15 and 60 days) and the results (S6 and S9 Tables) were consistent with the main results. Meanwhile, the limited availability of HPV typing data eliminated the possibility to explore more detailed insights into genotype-specific risks.

Second, HPV infection was defined as the first abnormal cytology result or the first positive HPV testing during follow-up, which does not account for the dynamic nature of HPV infection with possibly viral clearance and new incident HPV infections during follow-up. Additionally, this definition did not consider persistent HPV infection, a biologically and clinically different condition from transient infection, as the available follow-up may be inadequate to accurately distinguish between them. Consequently, our study may not fully capture the longitudinal trajectory of HPV infection status, which may result in nondifferential misclassification of exposure over time, potentially influencing the observed association between HPV infection and CVD in an unpredictable direction.

Third, women with HPV infection may engage more frequently with healthcare services, thereby increasing the likelihood of CVD diagnosis, particularly during the immediate time window after HPV infection. However, surveillance bias is likely minimal for the incidence of severe CVD subtypes, CVD-related mortality, and MACE. On the other hand, it is also possible that women experiencing early cardiovascular symptoms may seek medical care more frequently, increasing the likelihood of opportunistic cervical screening and HPV detection, which could introduce reverse causation and detection bias. To address this, we conducted a sensitivity analysis by excluding events that occurred within the first 3 months. The results showed that the associations remained unchanged (S6 and S9 Tables).

Fourth, due to the observational design of the study, the possibility of residual confounding not shared within full siblings, such as diet and physical activity, cannot be entirely ruled out. Fifth, information on smoking was available only from pregnancy visits recorded in the Medical Birth Registry [15], while smoking data from the general population were unavailable. This may have led to an underestimation of the number of smokers, and smoking patterns may differ between the preconceptional/during pregnancy period and the nonperinatal period. Sixth, since the study was conducted exclusively among women, the generalizability and broader interpretation to men were limited.

In conclusion, HPV infection was significantly associated with increased risk of incident CVD and CVD-related mortality, and the increased risk was independent of familial factors shared among full siblings. These associations were most pronounced within the first year after HPV infections and attenuated thereafter. Clinicians should be aware of the slightly short-term CVD risk in women with HPV; however, given the small absolute difference, women should not be unduly concerned.

## Supporting information

S1 Table International Classification of Diseases (ICD) codes for outcomes and covariates. (PDF)

S2 Table Study population characteristics in the sibling-matched cohort. Abbreviations: CVD, cardiovascular disease; HPV, human papillomavirus.
(PDF)

S3 Table Study population characteristics among four exposure groups in the population-matched cohort. Abbreviations: CVD, cardiovascular disease; CIN, cervical intraepithelial neoplasia; HPV, human papillomavirus.
(PDF)

S4 Table Study population characteristics among four exposure groups in the sibling-matched cohort. Abbreviations: CVD, cardiovascular disease; HPV, human papillomavirus.
(PDF)

S5 Table Subgroup analyses for any incident CVD associated with HPV infection in the population-matched cohort. Abbreviations: CVD, cardiovascular disease; HPV, human papillomavirus; HR, hazard ratio; CI, confidence interval; aARD, adjusted absolute rate difference; aPAF, adjusted population-attributable fraction; CIN3+, cervical intraepithelial neoplasia grade 3 or worse.
(PDF)

S6 Table Sensitivity analyses for any incident CVD associated with HPV infection in the population-matched cohort. Abbreviations: CVD, cardiovascular disease; HPV, human papillomavirus; CI, confidence interval; aARD, adjusted absolute rate difference; aPAF, adjusted population-attributable fraction.
(PDF)

S7 Table Hazard ratios with 95% CIs for CVD-related mortality associated with HPV infection. Abbreviations: CI, confidence interval; CVD, cardiovascular disease; HPV, human papillomavirus; aARD, adjusted absolute rate difference; aPAF, adjusted population-attributable fraction.
(PDF)

S8 Table Subgroup analyses for overall CVD-related mortality associated with HPV infection in the population-matched cohort. Abbreviations: CVD, cardiovascular disease; HPV, human papillomavirus; HR, hazard ratio; CI, confidence interval; aARD, adjusted absolute rate difference; aPAF, adjusted population-attributable fraction; CIN3+, cervical intraepithelial neoplasia grade 3 or worse.
(PDF)

S9 Table Sensitivity analyses for overall CVD-related mortality associated with HPV infection in the population-matched cohort. Abbreviations: CVD, cardiovascular disease; HPV, human papillomavirus; CI, confidence interval; aARD, adjusted absolute rate difference; aPAF, adjusted population-attributable fraction.
(PDF)

S10 Table Hazard ratios with 95% CIs for MACE associated with HPV infection. Abbreviations: CI, confidence interval; MACE, major adverse cardiovascular event; HPV, human papillomavirus; aARD, adjusted absolute rate difference; aPAF, adjusted population-attributable fraction.
(PDF)

S11 Table Mental disorders occurring after follow-up start among those without pervious mental disorders, by follow-up year, in the population-matched cohorts. (PDF)

S1 Fig Directed acyclic graph for exposure, outcome, and covariates. Abbreviations: HPV, human papillomavirus; CVD, cardiovascular disease; T2D, type 2 diabetes; COPD, chronic obstructive pulmonary disease; CIN3+, cervical intraepithelial neoplasia grade 3 or higher.
(TIF)

S1 File Research Plan. (DOCX)

S1 Checklist The checklist is originated from the STROBE Statement available at https://www.equator-network.org/reporting-guidelines/strobe/. (DOCX)

## Abbreviations

ARDs: absolute rate differences
CIs: confidence intervals
COPD: chronic obstructive pulmonary disease
CVD: cardiovascular disease
HDL: high-density lipoprotein
HPV: human papillomavirus
HRs: hazard ratios
ICD: International Classification of Diseases
IL-1β: interleukin-1β
IQR: interquartile range
IRs: incidence rates
LDL: low-density lipoprotein
MACE: major adverse cardiovascular event
NKCx: Swedish National Cervical Screening Registry
PAFs: population-attributable fractions
STROBE: Strengthening the Reporting of Observational Studies in Epidemiology
T2D: type 2 diabetes
TNF-α: tumor necrosis factor-alpha
VLDL: very-low-density lipoprotein.
