# Supplementary material for: Human papillomavirus infection and risk of cardiovascular disease: A population-based matched cohort study with sibling analysis
Source: PLoS Med. 2026 Sep 1;23(9):e1005222. doi: 10.1371/journal.pmed.1005222 (PMC13533340; doi:10.1371/journal.pmed.1005222)
Supplement: Supplementary file 1 [file pmed.1005222.s001.pdf]

S1 Table. International Classification of Diseases (ICD) codes for outcomes and covariates

Disease	ICD-10 (Used year: 1997-)	ICD-9 (Used year: 1987-1996)	ICD-8 (Used year: 1969-1986)
**Outcome**			
Any cardiovascular disease	I00-I74	391-438, 440-444	390-438, 440-444
Ischemic heart disease	I20-I25	410-414	410-414
Cerebrovascular disease	I60-I69	430-434, 436-438	430-434, 436-438
Emboli and thrombosis	I26, I74	415, 444	415, 444
Hypertensive disease	I10-I16, I674	401-405	401-405
Heart failure	I255, I420, I428, I429, I50	428	428
Arrhythmia/conduction disorder	I45-I49	426, 427, 427F	426, 427, 427F
**Covariate**			
Obesity	E65, E66	278A, 278B	277
Type 2 diabetes	E11	250	250
Hyperlipidemia	E78	272	279
Chronic obstructive pulmonary disease	J41- J44	491, 492, 496	491, 492
Cancers other than cervical cancer	C00-C75 (except for C53)	140-195 (except for 180)	140-195 (except for 180)
Cervical cancer	C53	180	180
Mental disorders	F10-F73, F78, F79, F84, F90	291, 292, 295-311, 314, 317-319	-
