# Supplementary material for: Human papillomavirus infection and risk of cardiovascular disease: A population-based matched cohort study with sibling analysis
Source: PLoS Med. 2026 Sep 1;23(9):e1005222. doi: 10.1371/journal.pmed.1005222 (PMC13533340; doi:10.1371/journal.pmed.1005222)
Supplement: Supplementary file 2 [file pmed.1005222.s002.pdf]

S2 Table. Study population characteristics in the sibling-matched cohort

Variable [a]	Total	Matched group	Exposed group
Number	318,424	174,637	143,787
Median age at index date, years	31.24 [26.04, 41.23]	31.75 [26.11, 41.65]	30.66 [25.98, 40.71]
Median follow-up year for incident CVD	7.11 [3.58, 11.65]	7.21 [3.64, 11.73]	6.93 [3.46, 11.52]
Median follow-up year for CVD-related mortality	7.36 [3.71, 11.98]	7.61 [3.78, 12.11]	7.24 [3.64, 11.88]
Birth country			
Sweden	302,303 (94.94%)	165,110 (94.54%)	137,193 (95.41%)
Others	16,121 (5.06%)	9,527 (5.46%)	6,594 (4.59%)
Residence county			
Stockholm	68,451 (21.50%)	37,045 (21.21%)	31,406 (21.84%)
Others	249,173 (78.25%)	137,393 (78.67%)	111,780 (77.74%)
Missing	800 (0.25%)	199 (0.11%)	601 (0.42%)
Highest education level			
Low (less than high school)	24,255 (7.62%)	15,532 (8.89%)	8,723 (6.07%)
Medium (high school)	138,055 (43.36%)	75,234 (43.08%)	62,821 (43.69%)
High (university or above)	153,709 (48.27%)	81,734 (46.80%)	71,975 (50.06%)
Missing	2,405 (0.76%)	2,137 (1.22%)	268 (0.19%)
Annual household income level [b]			
Low	130,471 (40.97%)	61,440 (35.18%)	69,031 (48.01%)
Medium	96,662 (30.36%)	55,433 (31.74%)	41,229 (28.67%)
High	88,607 (27.83%)	56,050 (32.10%)	32,557 (22.64%)
Missing	2,684 (0.84%)	1,714 (0.98%)	970 (0.67%)
High parity (>4)			
No	155,605 (48.87%)	91,134 (52.18%)	64,471 (44.84%)
Yes	2,153 (0.68%)	1,261 (0.72%)	892 (0.62%)
Nulliparity	160,666 (50.46%)	82,242 (47.09%)	78,424 (54.54%)
Smoking during pregnancy			
No	118,250 (37.14%)	70,842 (40.57%)	47,408 (32.97%)
Yes	27,857 (8.75%)	14,763 (8.45%)	13,094 (9.11%)
Nulliparity	160,666 (50.46%)	82,242 (47.09%)	78,424 (54.54%)
Missing	11,651 (3.66%)	6,790 (3.89%)	4,861 (3.38%)
Obesity [c]	423 (0.13%)	228 (0.13%)	195 (0.14%)
Type 2 diabetes [c]	1,680 (0.53%)	959 (0.55%)	721 (0.50%)
Hyperlipidemia [c]	736 (0.23%)	408 (0.23%)	328 (0.23%)
Chronic obstructive pulmonary disease [c]	931 (0.29%)	515 (0.29%)	416 (0.29%)
Cancers other than cervical cancer [c]	7,283 (2.29%)	3,893 (2.23%)	3,390 (2.36%)
Mental disorders [c]	61,504 (19.32%)	32,075 (18.37%)	29,429 (20.47%)
HPV vaccination [c]	54,781 (17.20%)	29,802 (17.07%)	24,979 (17.37%)

CVD, cardiovascular disease; HPV, human papillomavirus.

[a]: The characteristics were reported as median [interquartile range] for continuous variables and as frequency (percentage) for categorical variables.

[b]: Annual household income level was categorized into low, medium, and high based on the income level tertiles of the population aged 20 to 65.

[c]: For binary variables with two levels ("yes" and "no"), only the "yes" category was displayed.
