# Supplementary material for: Human papillomavirus infection and risk of cardiovascular disease: A population-based matched cohort study with sibling analysis
Source: PLoS Med. 2026 Sep 1;23(9):e1005222. doi: 10.1371/journal.pmed.1005222 (PMC13533340; doi:10.1371/journal.pmed.1005222)
Supplement: Supplementary file 3 [file pmed.1005222.s003.pdf]

S3 Table. Study population characteristics among four exposure groups in the population-matched cohort

Variable [a]	Group 1	Group 2	Group 3	Group 4
Number	21,384	139,445	323,872	12,744
Median age at index date, years	43.44 [30.25, 57.70]	29.67 [24.62, 37.91]	32.59 [27.00, 43.45]	25.18 [23.39, 30.89]
Median follow-up year for incident CVD	9.69 [4.69, 14.29]	5.16 [3.16, 7.32]	6.92 [2.97, 11.96]	1.23 [0.50, 2.22]
Median follow-up year for CVD-related mortality	11.56 [6.58, 15.65]	5.23 [3.25, 7.55]	7.32 [3.12, 12.32]	1.24 [0.50, 2.24]
Birth country				
Sweden	16,293 (76.19%)	109,176 (78.29%)	265,615 (82.01%)	8,508 (66.76%)
Others	5,091 (23.81%)	30,269 (21.71%)	58,257 (17.99%)	4,236 (33.24%)
Residence county				
Stockholm	3,206 (14.99%)	35,948 (25.78%)	70,906 (21.89%)	3,882 (30.46%)
Others	17,532 (81.99%)	99,304 (71.21%)	249,283 (76.97%)	8,360 (65.60%)
Missing	646 (3.02%)	4,193 (3.01%)	3,683 (1.14%)	502 (3.94%)
Highest education level				
Low (less than high school)	3,424 (16.01%)	10,022 (7.19%)	24,065 (7.43%)	979 (7.68%)
Medium (high school)	9,433 (44.11%)	57,582 (41.29%)	132,824 (41.01%)	4,857 (38.11%)
High (university or above)	7,325 (34.25%)	64,313 (46.12%)	159,699 (49.31%)	5,571 (43.71%)
Missing	1,202 (5.62%)	7,528 (5.40%)	7,284 (2.25%)	1,337 (10.49%)
Annual household income level [b]				
Low	9,591 (44.85%)	72,825 (52.22%)	151,832 (46.88%)	7,868 (61.74%)
Medium	6,359 (29.74%)	36,011 (25.82%)	97,669 (30.16%)	2,179 (17.10%)
High	4,595 (21.49%)	25,503 (18.29%)	68,678 (21.21%)	2,026 (15.90%)
Missing	839 (3.92%)	5,106 (3.66%)	5,693 (1.76%)	671 (5.27%)
High parity (>4)				
No	10,675 (49.92%)	47,422 (34.01%)	150,567 (46.49%)	1,275 (10.00%)
Yes	332 (1.55%)	652 (0.47%)	2,116 (0.65%)	25 (0.20%)
Nulliparity	10,377 (48.53%)	91,371 (65.52%)	171,189 (52.86%)	11,444 (89.80%)
Smoking during pregnancy				
No	5,783 (27.04%)	34,085 (24.44%)	108,635 (33.54%)	725 (5.69%)
Yes	2,421 (11.32%)	11,103 (7.96%)	31,911 (9.85%)	457 (3.59%)
Nulliparity	10,377 (48.53%)	91,371 (65.52%)	171,189 (52.86%)	11,444 (89.80%)
Missing	2,803 (13.11%)	2,886 (2.07%)	12,137 (3.75%)	118 (0.93%)
Obesity [c]	90 (0.42%)	111 (0.08%)	412 (0.13%)	6 (0.05%)
Type 2 diabetes [c]	275 (1.29%)	660 (0.47%)	1,853 (0.57%)	36 (0.28%)
Hyperlipidemia [c]	75 (0.35%)	273 (0.20%)	871 (0.27%)	23 (0.18%)
Chronic obstructive pulmonary disease [c]	173 (0.81%)	365 (0.26%)	1,078 (0.33%)	27 (0.21%)
Cancers other than cervical cancer [c]	1,586 (7.42%)	2,411 (1.73%)	8,095 (2.50%)	112 (0.88%)
Mental disorders [c]	3,529 (16.50%)	30,899 (22.16%)	65,102 (20.10%)	2,954 (23.18%)
Maternal history of CIN3+ [c]	983 (4.60%)	7,855 (5.63%)	19,319 (5.97%)	601 (4.72%)
Parental history of CVD [c]	7,428 (34.74%)	38,581 (27.67%)	103,509 (31.96%)	2,493 (19.56%)
HPV vaccination [c]	555 (2.60%)	32,858 (23.56%)	43,020 (13.28%)	6,042 (47.41%)

CVD, cardiovascular disease; CIN, cervical intraepithelial neoplasia; HPV, human papillomavirus.

[a]: The characteristics were reported as median [interquartile range] for continuous variables and as frequency (percentage) for categorical variables.

[b]: Annual household income level was categorized into low, medium, and high based on the income level tertiles of the population aged 20 to 65.

[c]: For binary variables with two levels ("yes" and "no"), only the "yes" category was displayed.
