# Supplementary material for: Human papillomavirus infection and risk of cardiovascular disease: A population-based matched cohort study with sibling analysis
Source: PLoS Med. 2026 Sep 1;23(9):e1005222. doi: 10.1371/journal.pmed.1005222 (PMC13533340; doi:10.1371/journal.pmed.1005222)
Supplement: Supplementary file 4 [file pmed.1005222.s004.pdf]

S4 Table. Study population characteristics among four exposure groups in the sibling-matched cohort

Variable [a]	Group 1	Group 2	Group 3	Group 4
Number	5,610	38,462	97,629	2,086
Median age at index date, years	41.96 [29.10, 53.87]	28.74 [24.33, 35.85]	31.45 [26.45, 41.66]	24.63 [23.35, 28.20]
Median follow-up year for incident CVD	10.81 [5.99, 14.93]	5.58 [3.64, 7.88]	8.18 [3.47, 12.76]	1.23 [0.50, 2.39]
Median follow-up year for CVD-related mortality	12.19 [7.66, 16.12]	5.67 [3.70, 7.95]	8.66 [3.66, 13.11]	1.24 [0.50, 2.50]
Birth country				
Sweden	5,323 (94.88%)	36,501 (94.90%)	93,461 (95.73%)	1,908 (91.47%)
Others	287 (5.12%)	1,961 (5.10%)	4,168 (4.27%)	178 (8.53%)
Residence county				
Stockholm	771 (13.74%)	10,012 (26.03%)	20,036 (20.52%)	587 (28.14%)
Others	4,798 (85.53%)	28,279 (73.52%)	77,226 (79.10%)	1,477 (70.81%)
Missing	41 (0.73%)	171 (0.44%)	367 (0.38%)	22 (1.05%)
Highest education level				
Low (less than high school)	740 (13.19%)	2,164 (5.63%)	5,657 (5.79%)	162 (7.77%)
Medium (high school)	2,798 (49.88%)	17,025 (44.26%)	42,039 (43.06%)	959 (45.97%)
High (university or above)	2,061 (36.74%)	19,187 (49.89%)	49,773 (50.98%)	954 (45.73%)
Missing	11 (0.20%)	86 (0.22%)	160 (0.16%)	11 (0.53%)
Annual household income level [b]				
Low	2,321 (41.37%)	20,017 (52.04%)	45,378 (46.48%)	1,315 (63.04%)
Medium	1,686 (30.05%)	10,038 (26.10%)	29,165 (29.87%)	340 (16.30%)
High	1,533 (27.33%)	8,159 (21.21%)	22,466 (23.01%)	399 (19.13%)
Missing	70 (1.25%)	248 (0.64%)	620 (0.64%)	32 (1.53%)
High parity (>4)				
No	3,238 (57.72%)	14,046 (36.52%)	46,944 (48.08%)	243 (11.65%)
Yes	85 (1.52%)	180 (0.47%)	619 (0.63%)	8 (0.38%)
Nulliparity	2,287 (40.77%)	24,236 (63.01%)	50,066 (51.28%)	1,835 (87.97%)
Smoking during pregnancy				
No	1,850 (32.98%)	10,463 (27.20%)	34,962 (35.81%)	133 (6.38%)
Yes	752 (13.40%)	2,999 (7.80%)	9,247 (9.47%)	96 (4.60%)
Nulliparity	2,287 (40.77%)	24,236 (63.01%)	50,066 (51.28%)	1,835 (87.97%)
Missing	721 (12.85%)	764 (1.99%)	3,354 (3.44%)	22 (1.05%)
Obesity [c]	21 (0.37%)	36 (0.09%)	135 (0.14%)	3 (0.14%)
Type 2 diabetes [c]	54 (0.96%)	163 (0.42%)	502 (0.51%)	2 (0.10%)
Hyperlipidemia [c]	15 (0.27%)	75 (0.19%)	234 (0.24%)	4 (0.19%)
Chronic obstructive pulmonary disease [c]	30 (0.53%)	102 (0.27%)	276 (0.28%)	8 (0.38%)
Cancers other than cervical cancer [c]	351 (6.26%)	720 (1.87%)	2,303 (2.36%)	16 (0.77%)
Mental disorders [c]	991 (17.66%)	8,849 (23.01%)	19,004 (19.47%)	585 (28.04%)
HPV vaccination [c]	193 (3.44%)	10,158 (26.41%)	13,396 (13.72%)	1,232 (59.06%)

CVD, cardiovascular disease; HPV, human papillomavirus.

[a]: The characteristics were reported as median [interquartile range] for continuous variables and as frequency (percentage) for categorical variables.

[b]: Annual household income level was categorized into low, medium, and high based on the income level tertiles of the population aged 20 to 65.

[c]: For binary variables with two levels (“yes” and “no”), only the “yes” category was displayed.
