# Supplementary material for: Human papillomavirus infection and risk of cardiovascular disease: A population-based matched cohort study with sibling analysis
Source: PLoS Med. 2026 Sep 1;23(9):e1005222. doi: 10.1371/journal.pmed.1005222 (PMC13533340; doi:10.1371/journal.pmed.1005222)
Supplement: Supplementary file 5 [file pmed.1005222.s005.pdf]

S5 Table. Subgroup analyses for any incident CVD associated with HPV infection in the population matched cohort

Group	Number of events		Incidence [95% CI], per 1,000 person-years		HR [95% CI]	aARD [95% CI]	aPAF [95% CI], %	P for interaction
	Matched group	Exposed group	Matched group	Exposed group	Model 2 [a]			
**Overall**	128,373	28,793	7.93 [7.88, 7.97]	8.27 [8.18, 8.37]	1.07 [1.05, 1.08]	0.53 [0.42, 0.65]	1.16 [0.93, 1.38]	
**By index age groups**								
<50 years old	71,923	16,269	5.10 [5.06, 5.14]	5.31 [5.23, 5.39]	1.02 [1.00, 1.04]	0.11 [0.02, 0.21]	0.40 [0.09, 0.72]	<0.001
≥50 years old	56,450	12,524	26.93 [26.71, 27.16]	30.20 [29.67, 30.73]	1.13 [1.11, 1.16]	3.60 [2.98, 4.22]	2.14 [1.81, 2.47]	
**By exposure types**								
Cytology+	128,373	18,859	7.93 [7.88, 7.97]	8.92 [8.79, 9.05]	1.08 [1.06, 1.10]	0.61 [0.47, 0.75]	0.92 [0.73, 1.12]	0.063
HPV testing+	128,373	9,934	7.93 [7.88, 7.97]	7.27 [7.13, 7.41]	1.05 [1.02, 1.07]	0.38 [0.19, 0.56]	0.33 [0.17, 0.48]	
**By comorbidities**								
Yes	34,239	8,604	12.03 [11.91, 12.16]	12.47 [12.21, 12.73]	1.07 [1.03, 1.11]	0.84 [0.35, 1.32]	1.31 [0.60, 2.01]	0.113
No	94,134	20,189	7.05 [7.01, 7.10]	7.23 [7.14, 7.33]	1.06 [1.04, 1.08]	0.41 [0.29, 0.53]	0.98 [0.71, 1.25]	
**By mental disorders**								
Yes	24,359	6,190	9.90 [9.78, 10.03]	10.18 [9.93, 10.44]	1.04 [0.99, 1.10]	0.43 [-0.08, 0.94]	0.84 [-0.12, 1.81]	0.128
No	104,014	22,603	7.57 [7.53, 7.62]	7.87 [7.77, 7.97]	1.08 [1.06, 1.09]	0.57 [0.44, 0.69]	1.25 [0.99, 1.50]	
**By maternal history of CIN3+**								
Yes	5,349	1,585	7.68 [7.47, 7.89]	7.65 [7.28, 8.04]	1.00 [0.84, 1.20]	0.03 [-1.35, 1.41]	0.08 [-4.00, 4.17]	0.600
No	123,024	27,208	7.94 [7.89, 7.98]	8.31 [8.21, 8.41]	1.07 [1.05, 1.08]	0.53 [0.41, 0.64]	1.13 [0.90, 1.36]	
**By parental history of CVD**								
Yes	56,336	12,689	11.79 [11.69, 11.89]	12.35 [12.14, 12.57]	1.08 [1.06, 1.11]	0.99 [0.68, 1.29]	1.42 [1.01, 1.83]	0.003
No	72,037	16,104	6.31 [6.26, 6.35]	6.56 [6.46, 6.67]	1.05 [1.03, 1.07]	0.31 [0.18, 0.43]	0.84 [0.51, 1.18]	
**By HPV vaccination**								
Yes	3,684	811	2.78 [2.69, 2.87]	2.71 [2.53, 2.90]	0.99 [0.88, 1.11]	-0.03 [-0.32, 0.27]	-0.19 [-2.41, 2.02]	0.221
No	124,689	27,982	8.38 [8.34, 8.43]	8.80 [8.69, 8.90]	1.07 [1.06, 1.09]	0.59 [0.46, 0.72]	1.24 [0.99, 1.49]	

CVD, cardiovascular disease; HPV, human papillomavirus; HR, hazard ratio; CI, confidence interval; aARD, adjusted absolute rate difference; aPAF, adjusted population-attributable fraction; CIN3+, cervical intraepithelial neoplasia grade 3 or worse.

[a]: Adjusted for the matching variable, birth country, residence county, education, annual family income, high parity, HPV vaccination, obesity, type 2 diabetes, hyperlipidemia, chronic obstructive pulmonary disease, cancers other than cervical cancer, mental disorders, maternal history of CIN3+, and parental history of CVD (except for the stratified variable).
