# Supplementary material for: Human papillomavirus infection and risk of cardiovascular disease: A population-based matched cohort study with sibling analysis
Source: PLoS Med. 2026 Sep 1;23(9):e1005222. doi: 10.1371/journal.pmed.1005222 (PMC13533340; doi:10.1371/journal.pmed.1005222)
Supplement: Supplementary file 6 [file pmed.1005222.s006.pdf]

S6 Table. Sensitivity analyses for any incident CVD associated with HPV infection in the population matched cohort

	Number of events		Incidence [95% CI], per 1,000 person-years		Hazard ratio [95% CI]			aARD [95% CI]	aPAF [95% CI], %
	Matched group	Exposed group	Matched group	Exposed group	Model 1 [a]	Model 2 [b]	Model 3 [c]		
Restricting to women without follow-up cervical cancer	128,330	28,361	7.92 [7.88, 7.97]	8.21 [8.11, 8.30]	1.09 [1.07, 1.10]	1.06 [1.05, 1.08]		0.49 [0.38, 0.60]	1.05 [0.83, 1.28]
Restricting to primary diagnosed cardiovascular disease cases	67,432	15,094	4.09 [4.06, 4.12]	4.26 [4.19, 4.32]	1.07 [1.05, 1.09]	1.06 [1.04, 1.07]		0.23 [0.15, 0.30]	0.95 [0.64, 1.27]
Restricting to women with parity ≥1	82,236	17,953	10.17 [10.10, 10.24]	11.22 [11.06, 11.38]	1.09 [1.07, 1.11]	1.05 [1.03, 1.07]	1.04 [1.03, 1.06]	0.49 [0.30, 0.68]	0.83 [0.52, 1.13]
Excluding CVD events occurring within the first 3 follow-up months	124,860	27,329	7.71 [7.67, 7.75]	7.85 [7.76, 7.95]	1.07 [1.05, 1.08]	1.04 [1.03, 1.05]		0.31 [0.20, 0.42]	0.70 [0.46, 0.93]
Using a 15-day cut-off for the intervals between cytology and HPV testing to define HPV infection	128,491	28,831	7.93 [7.88, 7.97]	8.28 [8.18, 8.37]	1.09 [1.08, 1.11]	1.07 [1.05, 1.08]		0.54 [0.42, 0.65]	1.16 [0.93, 1.39]
Using a 60-day cut-off for the intervals between cytology and HPV testing to define HPV infection	128,056	28,728	7.92 [7.88, 7.96]	8.27 [8.17, 8.36]	1.09 [1.08, 1.11]	1.07 [1.05, 1.08]		0.54 [0.43, 0.65]	1.17 [0.94, 1.39]

CVD, cardiovascular disease; HPV, human papillomavirus; CI, confidence interval; aARD, adjusted absolute rate difference; aPAF, adjusted population-attributable fraction.

[a]: Crude model.

[b]: Adjusted for the matching variable, birth country, residence county, education, annual family income, high parity, HPV vaccination, obesity, type 2 diabetes, hyperlipidemia, chronic obstructive pulmonary disease, cancers other than cervical cancer, mental disorders, maternal history of cervical intraepithelial neoplasia grade 3 or worse, and parental history of CVD.

[c]: Further adjusted for smoking during pregnancy based on the covariates included in the Model 2. This model was only used when restricting the analysis to women with parity ≥1 because smoking data are available only for women with a history of pregnancy from the Medical Birth Registry in our datasets.
