# Supplementary material for: Human papillomavirus infection and risk of cardiovascular disease: A population-based matched cohort study with sibling analysis
Source: PLoS Med. 2026 Sep 1;23(9):e1005222. doi: 10.1371/journal.pmed.1005222 (PMC13533340; doi:10.1371/journal.pmed.1005222)
Supplement: Supplementary file 7 [file pmed.1005222.s007.pdf]

S7 Table. Hazard ratios with 95% CIs for CVD-related mortality associated with HPV infection

Design and outcome	Number of events		Mortality [95% CI], per 100,000 person-years		Hazard ratio [95% CI]		aARD [95% CI]	aPAF [95% CI], %
	Matched group	Exposed group	Matched group	Exposed group	Model 1 [a]	Model 2 [b]		
**Population matched cohort**								
**Total follow-up time**								
Any CVD	4,941	1,221	29.39 [28.59, 30.23]	33.73 [31.89, 35.67]	1.34 [1.25, 1.44]	1.25 [1.16, 1.34]	6.71 [4.24, 9.18]	4.12 [2.88, 5.35]
Ischemic heart disease	944	188	5.63 [5.28, 6.00]	5.20 [4.51, 6.00]	1.11 [0.94, 1.32]	1.01 [0.84, 1.22]	0.05 [-0.89, 0.99]	0.18 [-3.16, 3.53]
Cerebrovascular disease	878	244	5.23 [4.90, 5.59]	6.75 [5.96, 7.66]	1.44 [1.24, 1.68]	1.38 [1.17, 1.62]	1.97 [0.81, 3.12]	6.10 [3.41, 8.79]
Emboli and thrombosis	450	147	2.68 [2.45, 2.94]	4.07 [3.46, 4.78]	1.74 [1.43, 2.12]	1.74 [1.40, 2.15]	1.93 [0.96, 2.89]	11.07 [7.49, 14.64]
Hypertensive disease	357	73	2.13 [1.92, 2.36]	2.02 [1.61, 2.54]	1.25 [0.94, 1.67]	1.19 [0.88, 1.61]	0.35 [-0.32, 1.01]	2.96 [-1.83, 7.74]
Heart failure	542	138	3.23 [2.97, 3.52]	3.82 [3.23, 4.51]	1.28 [1.04, 1.59]	1.19 [0.95, 1.50]	0.59 [-0.26, 1.45]	3.05 [-0.67, 6.77]
Arrhythmia/conduction disorder	1,151	258	6.86 [6.48, 7.27]	7.14 [6.32, 8.07]	1.28 [1.10, 1.49]	1.16 [0.99, 1.36]	0.96 [-0.15, 2.08]	2.75 [-0.02, 5.52]
Other CVDs	619	173	3.69 [3.41, 3.99]	4.79 [4.13, 5.56]	1.47 [1.23, 1.77]	1.33 [1.09, 1.62]	1.16 [0.22, 2.10]	5.58 [2.09, 9.07]
**<1-year follow-up time**								
Any CVD	265	97	11.15 [9.89, 12.58]	19.54 [16.01, 23.84]	2.00 [1.57, 2.56]	1.86 [1.41, 2.45]	8.78 [3.51, 14.06]	13.18 [8.34, 18.03]
Ischemic heart disease	50	12	2.10 [1.59, 2.78]	2.42 [1.37, 4.26]	1.22 [0.63, 2.35]	0.92 [0.40, 2.11]	-0.17 [-1.77, 1.43]	-1.67 [-19.08, 15.74]
Cerebrovascular disease	52	14	2.19 [1.67, 2.87]	2.82 [1.67, 4.76]	1.43 [0.77, 2.65]	1.37 [0.68, 2.76]	0.76 [-1.20, 2.73]	6.07 [-5.71, 17.86]
Emboli and thrombosis	33	26	1.39 [0.99, 1.95]	5.24 [3.57, 7.69]	4.15 [2.44, 7.06]	5.35 [2.64, 10.85]	5.95 [0.77, 11.12]	36.96 [24.69, 49.23]
Hypertensive disease	7	5	0.29 [0.14, 0.62]	1.01 [0.42, 2.42]	4.08 [1.25, 13.39]	5.39 [0.51, 56.61]	1.20 [-2.27, 4.67]	37.02 [5.92, 68.13]
Heart failure	30	8	1.26 [0.88, 1.81]	1.61 [0.81, 3.22]	2.86 [1.20, 6.81]	2.05 [0.58, 7.28]	0.67 [-0.99, 2.33]	18.67 [-5.99, 43.32]
Arrhythmia/conduction disorder	65	19	2.74 [2.15, 3.49]	3.83 [2.44, 6.00]	1.78 [1.02, 3.09]	1.36 [0.65, 2.86]	0.79 [-1.41, 3.00]	6.96 [-7.54, 21.46]
Other CVDs	28	13	1.18 [0.81, 1.71]	2.62 [1.52, 4.51]	1.52 [0.77, 2.99]	1.01 [0.43, 2.38]	0.01 [-1.40, 1.43]	0.21 [-19.67, 20.08]
**≥1-year follow-up time**								
Any CVD	4,676	1,124	32.40 [31.48, 33.34]	35.81 [33.78, 37.97]	1.30 [1.21, 1.40]	1.21 [1.12, 1.30]	6.35 [3.58, 9.12]	3.53 [2.24, 4.81]
Ischemic heart disease	894	176	6.21 [5.81, 6.63]	5.62 [4.85, 6.51]	1.10 [0.92, 1.32]	1.01 [0.83, 1.22]	0.04 [-1.03, 1.11]	0.12 [-3.34, 3.58]
Cerebrovascular disease	826	230	5.73 [5.36, 6.14]	7.34 [6.45, 8.36]	1.44 [1.23, 1.69]	1.38 [1.17, 1.63]	2.19 [0.87, 3.50]	6.11 [3.34, 8.89]
Emboli and thrombosis	417	121	2.90 [2.63, 3.19]	3.86 [3.23, 4.62]	1.53 [1.24, 1.90]	1.51 [1.19, 1.90]	1.43 [0.45, 2.42]	8.00 [4.12, 11.88]
Hypertensive disease	350	68	2.43 [2.19, 2.70]	2.17 [1.71, 2.75]	1.18 [0.88, 1.58]	1.12 [0.82, 1.53]	0.26 [-0.49, 1.00]	1.91 [-3.07, 6.88]
Heart failure	512	130	3.56 [3.26, 3.88]	4.15 [3.50, 4.93]	1.23 [0.98, 1.54]	1.16 [0.91, 1.47]	0.56 [-0.42, 1.53]	2.48 [-1.32, 6.28]
Arrhythmia/conduction disorder	1,086	239	7.54 [7.10, 8.00]	7.63 [6.72, 8.66]	1.25 [1.07, 1.46]	1.14 [0.97, 1.35]	0.98 [-0.30, 2.25]	2.48 [-0.36, 5.33]
Other CVDs	591	160	4.10 [3.79, 4.45]	5.11 [4.38, 5.97]	1.47 [1.22, 1.78]	1.34 [1.09, 1.65]	1.33 [0.25, 2.41]	5.78 [2.20, 9.36]
**Sibling matched cohort**								
Any CVD	347	250	24.24 [21.82, 26.93]	21.73 [19.19, 24.60]	1.12 [0.95, 1.33]	1.25 [1.01, 1.53]	5.96 [0.24, 12.16]	8.26 [1.32, 15.21]

CI, confidence interval; CVD, cardiovascular disease; HPV, human papillomavirus; aARD, adjusted absolute rate difference; aPAF, adjusted population-attributable fraction.

[a]: Crude model.

[b]: In the population-matched cohort, adjustments were made for the matching variable, birth country, residence county, education, annual family income, high parity, HPV vaccination, obesity, type 2 diabetes, hyperlipidemia, chronic obstructive pulmonary disease, cancers other than cervical cancer, mental disorders, maternal history of cervical intraepithelial neoplasia grade 3 or worse, and parental history of CVD. In the sibling-matched cohort, adjustments included the same covariates as in the population-matched cohort, with the addition of birth year, but excluded maternal history of cervical intraepithelial neoplasia grade 3 or worse and parental history of CVD.
