# Supplementary material for: Human papillomavirus infection and risk of cardiovascular disease: A population-based matched cohort study with sibling analysis
Source: PLoS Med. 2026 Sep 1;23(9):e1005222. doi: 10.1371/journal.pmed.1005222 (PMC13533340; doi:10.1371/journal.pmed.1005222)
Supplement: Supplementary file 8 [file pmed.1005222.s008.pdf]

S8 Table. Subgroup analyses for overall CVD-related mortality associated with HPV infection in the population matched cohort

Group	Number of events		Mortality [95% CI], per 100,000 person-years		HR [95% CI]	aARD [95% CI]	aPAF [95% CI], %	P for interaction
	Matched group	Exposed group	Matched group	Exposed group	Model 2 [a]			
**Overall**	4,941	1,221	29.39 [28.59, 30.23]	33.73 [31.89, 35.67]	1.25 [1.16, 1.34]	6.71 [4.24, 9.18]	4.12 [2.88, 5.35]	
**By index age groups**								
<50 years old	1,337	339	9.26 [8.77, 9.77]	10.78 [9.69, 11.99]	1.21 [1.05, 1.39]	1.78 [0.31, 3.24]	3.71 [1.15, 6.27]	0.100
≥50 years old	3,604	882	152.20 [147.31, 157.25]	185.78 [173.92, 198.46]	1.26 [1.16, 1.37]	37.31 [21.92, 52.70]	4.26 [2.84, 5.68]	
**By exposure types**								
Cytology+	4,941	977	29.39 [28.59, 30.23]	43.94 [41.27, 46.79]	1.35 [1.24, 1.46]	9.48 [6.51, 12.45]	4.59 [3.49, 5.69]	<0.001
HPV testing+	4,941	244	29.39 [28.59, 30.23]	17.47 [15.41, 19.80]	0.92 [0.78, 1.09]	-2.22 [-6.41, 1.97]	-0.40 [-1.21, 0.42]	
**By comorbidities**								
Yes	2,017	538	67.35 [64.47, 70.35]	73.89 [67.91, 80.41]	1.25 [1.13, 1.38]	15.39 [7.42, 23.35]	4.55 [2.63, 6.47]	0.772
No	2,924	683	21.17 [20.41, 21.95]	23.62 [21.91, 25.46]	1.21 [1.10, 1.32]	4.20 [2.01, 6.39]	3.39 [1.91, 4.87]	
**By mental disorders**								
Yes	1,255	316	48.97 [46.33, 51.76]	49.83 [44.63, 55.64]	1.16 [0.87, 1.55]	7.10 [-7.91, 22.10]	2.98 [-2.46, 8.42]	0.272
No	3,686	905	25.87 [25.05, 26.72]	30.31 [28.40, 32.35]	1.25 [1.15, 1.36]	6.14 [3.58, 8.70]	4.16 [2.75, 5.57]	
**By maternal history of CIN3+**								
Yes	174	68	24.10 [20.78, 27.97]	31.66 [24.96, 40.16]	0.67 [0.05, 9.63]	-8.09 [-52.13, 35.95]	-13.43 [-122.30, 95.45]	0.200
No	4,767	1,153	29.63 [28.80, 30.48]	33.86 [31.96, 35.87]	1.24 [1.15, 1.34]	6.59 [4.04, 9.14]	3.98 [2.72, 5.25]	
**By parental history of CVD**								
Yes	2,300	534	45.59 [43.76, 47.49]	49.07 [45.08, 53.41]	1.14 [1.00, 1.29]	5.79 [-0.50, 12.08]	2.36 [-0.09, 4.62]	0.088
No	2,641	687	22.45 [21.61, 23.32]	27.13 [25.18, 29.24]	1.35 [1.21, 1.51]	7.27 [4.15, 10.40]	5.66 [3.81, 7.51]	
**By HPV vaccination**								
Yes	30	3	2.25 [1.57, 3.22]	0.99 [0.32, 3.08]	1.53 [0.34, 7.01]	1.20 [-4.04, 6.44]	3.16 [-6.48, 12.80]	0.052
No	4,911	1,218	31.73 [30.86, 32.63]	36.71 [34.70, 38.83]	1.19 [1.11, 1.27]	5.89 [3.35, 8.44]	3.11 [1.97, 4.26]	

CVD, cardiovascular disease; HPV, human papillomavirus; HR, hazard ratio; CI, confidence interval; aARD, adjusted absolute rate difference; aPAF, adjusted population-attributable fraction; CIN3+, cervical intraepithelial neoplasia grade 3 or worse.

[a]: Adjusted for the matching variable, birth country, residence county, education, annual family income, high parity, HPV vaccination, obesity, type 2 diabetes, hyperlipidemia, chronic obstructive pulmonary disease, cancers other than cervical cancer, mental disorders, maternal history of CIN3+, and parental history of CVD (except for the stratified variable).
