# Supplementary material for: Human papillomavirus infection and risk of cardiovascular disease: A population-based matched cohort study with sibling analysis
Source: PLoS Med. 2026 Sep 1;23(9):e1005222. doi: 10.1371/journal.pmed.1005222 (PMC13533340; doi:10.1371/journal.pmed.1005222)
Supplement: Supplementary file 9 [file pmed.1005222.s009.pdf]

S9 Table. Sensitivity analyses for overall CVD-related mortality associated with HPV infection in the population matched cohort

	Number of events		Mortality [95% CI], per 100,000 person-years		Hazard ratio [95% CI]			aARD [95% CI]	aPAF [95% CI], %
	Matched group	Exposed group	Matched group	Exposed group	Model 1 [a]	Model 2 [b]	Model 3 [c]		
Restricting to women without follow-up cervical cancer	4,935	1,178	29.36 [28.56, 30.19]	32.80 [30.98, 34.72]	1.31 [1.22, 1.41]	1.21 [1.13, 1.31]		5.84 [3.39, 8.28]	3.58 [2.33, 4.83]
Restricting to primary diagnosed cardiovascular disease cases	2,337	509	13.90 [13.35, 14.48]	14.06 [12.89, 15.34]	1.47 [1.34, 1.61]	1.35 [1.23, 1.49]		5.16 [3.26, 7.07]	5.81 [4.17, 7.45]
Restricting to women with parity ≥1	2,553	613	30.11 [28.97, 31.30]	36.33 [33.56, 39.32]	1.28 [1.16, 1.42]	1.15 [1.03, 1.29]	1.14 [1.02, 1.28]	4.14 [0.70, 7.58]	2.79 [0.77, 4.81]
Excluding CVD events occurring within the first 3 follow-up months	4,878	1,194	29.02 [28.22, 29.85]	32.98 [31.16, 34.91]	1.33 [1.24, 1.42]	1.23 [1.14, 1.32]		6.24 [3.80, 8.68]	3.88 [2.63, 5.13]
Using a 15-day cut-off for the intervals between cytology and HPV testing to define HPV infection	4,941	1,223	29.37 [28.57, 30.20]	33.76 [31.92, 35.71]	1.34 [1.25, 1.44]	1.25 [1.16, 1.34]		6.74 [4.26, 9.22]	4.12 [2.88, 5.35]
Using a 60-day cut-off for the intervals between cytology and HPV testing to define HPV infection	4,929	1,218	29.37 [28.57, 30.21]	33.70 [31.86, 35.65]	1.34 [1.25, 1.44]	1.24 [1.16, 1.34]		6.65 [4.18, 9.12]	4.07 [2.84, 5.31]

CVD, cardiovascular disease; HPV, human papillomavirus; CI, confidence interval; aARD, adjusted absolute rate difference; aPAF, adjusted population-attributable fraction.

[a]: Crude model.

[b]: Adjusted for the matching variable, birth country, residence county, education, annual family income, high parity, HPV vaccination, obesity, type 2 diabetes, hyperlipidemia, chronic obstructive pulmonary disease, cancers other than cervical cancer, mental disorders, maternal history of cervical intraepithelial neoplasia grade 3 or worse, and parental history of CVD.

[c]: Further adjusted for smoking during pregnancy based on the covariates included in the Model 2. This model was only used when restricting the analysis to women with parity ≥1 because smoking data are available only for women with a history of pregnancy from the Medical Birth Registry in our datasets.
