# Supplementary material for: Human papillomavirus infection and risk of cardiovascular disease: A population-based matched cohort study with sibling analysis
Source: PLoS Med. 2026 Sep 1;23(9):e1005222. doi: 10.1371/journal.pmed.1005222 (PMC13533340; doi:10.1371/journal.pmed.1005222)
Supplement: Supplementary file 10 [file pmed.1005222.s010.pdf]

S10 Table. Hazard ratios with 95% CIs for MACE associated with HPV infection

Design and outcome	Number of events		Rate [95% CI], per 1,000 person-years		Hazard ratio [95% CI]		aARD [95% CI]	aPAF [95% CI], %
	Matched group	Exposed group	Matched group	Exposed group	Model 1 [a]	Model 2 [b]		
**Population matched cohort**								
Total follow-up time	23,195	5,246	1.49 [1.47, 1.51]	1.57 [1.53, 1.61]	1.12 [1.09, 1.16]	1.07 [1.04, 1.11]	0.11 [0.06, 0.16]	1.28 [0.73, 1.83]
<1-year follow-up time	2,495	705	1.10 [1.06, 1.14]	1.49 [1.39, 1.61]	1.42 [1.30, 1.54]	1.33 [1.21, 1.45]	0.36 [0.23, 0.49]	5.41 [3.88, 6.94]
≥1-year follow-up time	20,483	4,541	1.54 [1.52, 1.56]	1.57 [1.53, 1.62]	1.10 [1.06, 1.14]	1.05 [1.02, 1.09]	0.08 [0.03, 0.14]	0.94 [0.35, 1.54]

CI, confidence interval; MACE, major adverse cardiovascular event; HPV, human papillomavirus; aARD, adjusted absolute rate difference; aPAF, adjusted population-attributable fraction.

[a]: Crude model.

[b]: Adjusted for the matching variable, birth country, residence county, education, annual family income, high parity, HPV vaccination, obesity, type 2 diabetes, hyperlipidemia, chronic obstructive pulmonary disease, cancers other than cervical cancer, mental disorders, maternal history of cervical intraepithelial neoplasia grade 3 or worse, and parental history of cardiovascular disease.
