# Supplementary material for: Human papillomavirus infection and risk of cardiovascular disease: A population-based matched cohort study with sibling analysis
Source: PLoS Med. 2026 Sep 1;23(9):e1005222. doi: 10.1371/journal.pmed.1005222 (PMC13533340; doi:10.1371/journal.pmed.1005222)
Supplement: Supplementary file 11 [file pmed.1005222.s011.pdf]

S11 Table. Mental disorders occurring after follow-up start among those without pervious mental disorders, by follow-up year, in the population-matched cohorts

	Total	Matched group	Exposed group
Number	2,433,136	2,038,175	394,961
Mental disorders [a]	159,400 (6.55%)	128,456 (6.30%)	30,944 (7.83%)
Follow-up year			
1	28,250 (11.75%)	22,576 (10.91%)	5,674 (16.96%)
2	23,283 (8.86%)	18,722 (8.43%)	4,561 (11.26%)
3	19,574 (8.63%)	15,869 (8.25%)	3,705 (10.74%)
4	16,485 (6.79%)	13,293 (6.50%)	3,192 (8.32%)
5	14,023 (7.97%)	11,401 (7.70%)	2,622 (9.42%)
6	11,770 (6.23%)	9,565 (6.03%)	2,205 (7.27%)
7	9,748 (6.10%)	7,891 (5.90%)	1,857 (7.12%)
8	8,041 (5.91%)	6,450 (5.67%)	1,591 (7.17%)
9	6,758 (6.01%)	5,449 (5.82%)	1,309 (6.95%)
10	5,597 (5.20%)	4,494 (5.04%)	1,103 (6.00%)
11	4,301 (4.31%)	3,403 (4.13%)	898 (5.13%)
12	3,406 (3.83%)	2,766 (3.78%)	640 (4.03%)
13	2,554 (3.26%)	2,065 (3.21%)	489 (3.48%)
14	1,984 (2.81%)	1,565 (2.70%)	419 (3.28%)
15	1,432 (2.41%)	1,184 (2.43%)	248 (2.31%)
16	1,022 (2.11%)	827 (2.09%)	195 (2.22%)
17	706 (1.46%)	567 (1.43%)	139 (1.55%)
18	369 (0.82%)	284 (0.78%)	85 (1.03%)
19	97 (0.24%)	85 (0.26%)	12 (0.16%)

[a]: Mental disorders were reported as frequency (percentage) and only the “yes” category was displayed.
