# Supplementary material for: Human papillomavirus infection and risk of cardiovascular disease: A population-based matched cohort study with sibling analysis
Source: PLoS Med. 2026 Sep 1;23(9):e1005222. doi: 10.1371/journal.pmed.1005222 (PMC13533340; doi:10.1371/journal.pmed.1005222)
Supplement: Supplementary file 12 [file pmed.1005222.s013.docx]

**Human papillomavirus infection and risk of cardiovascular disease: a longitudinal matched cohort study**

**Authors:** Yunyang Deng, Mark Clements, Chaonan Shen, Jonas F. Ludvigsson, Qing Shen, Jiayao Lei

**Version date:** February 18, 2025

# 1. Background

Cardiovascular diseases (CVDs) remain the leading cause of morbidity and mortality globally^1^. Although advances in the identification and management of traditional CVD risk factors, CVD continues to occur among individuals without established risk factors^2^. Human papillomavirus (HPV) is the most common sexually transmitted virus and is responsible for nearly all cervical cancers^3^. Recent observational studies have reported positive associations between HPV infection and CVD incidence and/or mortality^4,5^. However, to our knowledge, no population-based cohort study has investigated this association in Europe. Furthermore, no sibling-matched cohort study has been conducted to date, which could account for unmeasured familial confounding.

# 2. Research question

What are the associations of HPV infection with the incidence and mortality of CVD?

# 3. Data source and study population

This longitudinal matched cohort study will use nationwide Swedish population and healthcare registers, linked at the individual level through unique personal identity numbers^6^. Data sources will include the Swedish National Cervical Screening Registry (NKCx)^7^, National Patient Register^8^, Death Registers, Prescribed Drug Register^9^, National Cancer Registry^10^, Total Population Register^11^, Medical Birth Register^12^, Multi-Generation Register^13^, and Longitudinal Integration Database for Health Insurance and Labor Market Studies (LISA)^14^.

From registers, women born after 1932, aged ≥23 years, and with screening data (cytology or HPV testing) from 2006 to 2022 will be identified. The Multi-Generation Register provides comprehensive familial linkage for individuals born since 1932^13^. Therefore, women born after 1932 will be selected to enable sibling-matched cohort analyses and comparisons with population-matched cohorts. In Sweden, the NKCx has recorded screening data for women aged ≥23 years since 1969, achieving nearly complete coverage by 1995^7^. Hence, women aged ≥23 years during follow up will be selected to ensure they had at least one screening result. The National Patient Register has provided nationwide inpatient care data since 1987 and outpatient specialist care data since 2001^8^. However, diagnoses of CVD and other diseases were incomplete in our databases before 2005, restricting the study period to 2006-2022.

The entry date will be defined as January 1, 2006, or the date a woman turned 23 years, whichever occurred later. Women will be excluded for the following reasons: death, emigration, loss to follow-up, pre-existing CVD, CIN2+, or HPV infection before entry date; HPV infection before age 23; or having HPV infection or CVD after death, emigration, loss to follow-up, or the end date (31 December 2022). Within the source population, women with new abnormal screening results (cytology or HPV testing) during 2006-2022 will be identified. The first abnormal screening result during follow-up will be defined as the index date for exposed women.

In population-matched cohorts, for each exposed woman, five women without HPV infection or outcomes at the exposed woman’s index date will be randomly matched by birth year from the source population as unexposed controls. Additionally, to reduce residual confounding from shared genetic and early environmental factors, we will also perform sibling-matched analyses. Briefly, we will only include exposed/index women who had at least one full sibling unaffected by HPV infection or CVD before the index woman’s diagnosis. Each unexposed woman will be assigned a pseudo-index date corresponding to the index date of the exposed woman to whom they will be matched. Follow-up will begin on the index/pseudo-index date and continue until outcomes occurred, death, emigration, loss to follow-up, or 31 December 2022, whichever came first. Additionally, unexposed women will be censored upon developing abnormal screening results during the follow-up period.

# 4. Assessment of exposure, outcome, and covariates

Cytological and HPV testing results will be used as proxies for HPV infection. In the NKCx, cytological results were coded using the Systematized Nomenclature of Medicine (SNOMED) and categorized as normal or abnormal. HPV testing results were classified as either negative or positive for 14 high-risk HPV types (16, 18, 31, 33, 35, 39, 45, 51, 52, 56, 58, 59, 66, and 68). In this study, an HPV infection will be defined as either an abnormal cytological result or a positive HPV test. The date of the first abnormal screening result during follow up will be regarded as the index/pseudo-index date for included women.

For CVD incidence, the primary outcome will be the occurrence of any CVD. Secondary outcomes will be the incidence of specific CVD subtypes, including ischemic heart disease, cerebrovascular disease, embolism and thrombosis, hypertensive disease, heart failure, arrhythmia/conduction disorder, and other CVD^15^. For CVD mortality, the primary outcomes will be mortality from any CVD, with secondary outcomes of mortality from CVD subtypes. Incident CVD cases will be first identified from the National Patient Register using International Classification of Disease (ICD) codes. Women without CVD data in the National Patient Register but with CVD-related death records in the Death Registers will be also classified as incident CVD cases. Additionally, data on CVD mortality will be extracted from the Death Registers using ICD codes.

Covariates will include birth year, birth country, residence county, education, income, high parity, smoking during pregnancy, obesity, type 2 diabetes (T2D), hyperlipidemia, chronic obstructive pulmonary disease (COPD), cancers other than cervical cancer, mental disorders, maternal history of CIN3+, and parental history of CVD. Data on birth year, birth country (Sweden, others, or missing), and residence county before the index date will be obtained from the Total Population Register. Education and income data before the index date will be sourced from LISA, categorizing education as low (less than high school), medium (high school), or high (university or above) and income into tertiles. Missing data for residence county, education, and income will be grouped into an additional category. Information on high parity (>4) and smoking during pregnancy will be extracted from the Medical Birth Register. Data on obesity, T2D, hyperlipidemia, COPD, and mental disorders will be derived from the National Patient Register. Cancer diagnoses will be obtained from the National Patient Register. Biological parent identification will be achieved via the Multi-Generation Register. Maternal history of CIN3+ will be then retrieved from the National Cancer Register, and parental history of CVD will be sourced from the National Patient Register.

# 5. Statistical analysis

The index characteristics will be presented as mean±standard deviation for continuous variables and as frequency (percentage) for categorical variables. These characteristics will be displayed for the overall cohort, women with HPV infection, and their matched reference population. The T-test and chi-square test will be utilized to compare these characteristics between the exposed and matched groups, as applicable.

In population-matched analyses, we will estimate the average hazard ratios (HRs) and 95% confidence intervals (CIs) from Cox models stratified by the matching variable, using follow-up time as the underlying time scale. Two models will be used: (1) Adjusted model 1: adjusted for the matching variable; (2) Adjusted model 2: additionally adjusted for birth country, residence county, education, income, high parity, smoking during pregnancy, obesity, T2D, hyperlipidemia, COPD, cancers other than cervical cancer, mental disorders, maternal history of CIN3+, and parental history of CVD.

Moreover, subgroup analyses will be conducted based on follow-up time after the index/pseudo-index date (<1 year or ≥1 year), exposure types (cytology or HPV testing), history of comorbidity (obesity, T2D, hyperlipidemia, COPD, cancers other than cervical cancer, or mental disorders) (no or yes), maternal history of CIN3+ (no or yes), and parental history of CVD (no or yes).

Additionally, several sensitivity analyses will be considered: (1) applying different buffer periods (0.5, 1, and 3 years). Only outcomes occurring after the buffer period will be considered as a valid outcome; (2) restricting to women who did not develop cervical cancer during follow-up (after the index/pseudo-index date but before the occurrence of outcomes) to mitigate the potential impact of cervical cancer treatments; (3) restricting to women who did not develop follow-up CIN2+ to mitigate the effect of CIN2+ treatments; (4) restricting to CVD cases excluding hypertensive disease; and (5) restricting to CVD cases identified only through primary diagnosis.

In sibling-matched analyses, Cox models will be stratified by sibling set and adjusted for the same confounders as in population-matched analyses except for maternal history of CIN3+ and parental history of CVD. Due to the limited number of events, analyses for CVD subtypes, subgroup analyses, and sensitivity analyses will not be conducted in sibling-matched analyses.

Data management and analyses were carried out using SAS version 9.4. All statistical tests were two-sided, with statistical significance set at P<0.05.

# 6. Administrative information

This prospective research plan was finalized on February 18, 2025, and was used to guide the study design and analytical strategy. Statistical analyses and manuscript preparation were conducted between March and October 2025, during which several changes were further made, including refinement of the study population, exposure and outcome definitions, the main statistical analysis from Cox regression to flexible parametric models, and subgroup and sensitivity analyses. No data-driven changes to the analyses were made. The core study objectives remain consistent.

In addition, several changes to the analyses were made during peer review: (1) We extended the follow-up end year from 2022 to 2024 after receiving updated data during the revision period. We believe that the longer follow-up period and larger study population further strengthened the manuscript. (2) We added absolute rate differences and population-attributable fractions in response to reviewers’ comments, as we agree that absolute differences are as important as relative differences. (3) In response to a reviewer comment, we added Table S12, which partially supports our hypothesis that psychological stress following a positive HPV test may contribute to the elevated CVD risk during the early follow-up period. (4) We included HPV vaccination as a covariate and conducted subgroup analyses by HPV vaccination, as we agree that HPV vaccination is an important factor in the association. (5) We added Tables S3 and S4 to present the characteristics of the study population across the four exposure groups. This is important because the exposure definition was based on both HPV testing and cytology. (6) We added a sensitivity analysis excluding outcome events occurring within the first three months of follow-up to partially assess potential reverse causation. (7) We added a sensitivity analysis using two alternative cutoffs (15 and 60 days) to define HPV infection because the 30-day cutoff used to define HPV infection based on the interval between cytology and HPV testing in Exposure Groups 2 and 3 was arbitrary. These changes do not change the conclusions.

# References:

1.	World Health Organization (WHO). Cardiovascular diseases (CVDs). 2021. Available from: https://www.who.int/news-room/fact-sheets/detail/cardiovascular-diseases-(cvds).

2.	Wang L, Lei J, Wang R, Li K. Non-Traditional Risk Factors as Contributors to Cardiovascular Disease. *Rev Cardiovasc Med* 2023; **24**(5): 134.

3.	de Martel C, Plummer M, Vignat J, Franceschi S. Worldwide burden of cancer attributable to HPV by site, country and HPV type. *Int J Cancer* 2017; **141**(4): 664–70.

4.	Cheong HS, Chang Y, Kim Y, et al. Human papillomavirus infection and cardiovascular mortality: a cohort study. *Eur Heart J* 2024; **45**(12): 1072–82.

5.	Joo EJ, Chang Y, Kwon MJ, Cho A, Cheong HS, Ryu S. High-Risk Human Papillomavirus Infection and the Risk of Cardiovascular Disease in Korean Women. *Circ Res* 2019; **124**(5): 747–56.

6.	Ludvigsson JF, Otterblad-Olausson P, Pettersson BU, Ekbom A. The Swedish personal identity number: possibilities and pitfalls in healthcare and medical research. *Eur J Epidemiol* 2009; **24**(11): 659–67.

7.	Elfström KM, Sparén P, Olausson P, Almstedt P, Strander B, Dillner J. Registry-based assessment of the status of cervical screening in Sweden. *J Med Screen* 2016; **23**(4): 217–26.

8.	Ludvigsson JF, Andersson E, Ekbom A, et al. External review and validation of the Swedish national inpatient register. *BMC Public Health* 2011; **11**: 450.

9.	Wettermark B, Hammar N, Fored CM, et al. The new Swedish Prescribed Drug Register--opportunities for pharmacoepidemiological research and experience from the first six months. *Pharmacoepidemiol Drug Saf* 2007; **16**(7): 726–35.

10.	Barlow L, Westergren K, Holmberg L, Talbäck M. The completeness of the Swedish Cancer Register: a sample survey for year 1998. *Acta Oncol* 2009; **48**(1): 27–33.

11.	Ludvigsson JF, Almqvist C, Bonamy AK, et al. Registers of the Swedish total population and their use in medical research. *Eur J Epidemiol* 2016; **31**(2): 125–36.

12.	Cnattingius S, Ericson A, Gunnarskog J, Källén B. A quality study of a medical birth registry. *Scand J Soc Med* 1990; **18**(2): 143–8.

13.	Ekbom A. The Swedish Multi-generation Register. *Methods Mol Biol* 2011; **675**: 215–20.

14.	Ludvigsson JF, Svedberg P, Olén O, Bruze G, Neovius M. The longitudinal integrated database for health insurance and labour market studies (LISA) and its use in medical research. *Eur J Epidemiol* 2019; **34**(4): 423–37.

15.	Song H, Fang F, Arnberg FK, et al. Stress related disorders and risk of cardiovascular disease: population based, sibling controlled cohort study. *BMJ* 2019; **365**: l1255.
