# Supplementary material for: Human papillomavirus infection and risk of cardiovascular disease: A population-based matched cohort study with sibling analysis
Source: PLoS Med. 2026 Sep 1;23(9):e1005222. doi: 10.1371/journal.pmed.1005222 (PMC13533340; doi:10.1371/journal.pmed.1005222)
Supplement: Supplementary file 14 [file pmed.1005222.s015.docx]

**Response to comments on manuscript PMEDICINE-D-25-03596R1 entitled “Human papillomavirus infection and risk of cardiovascular disease: a population-based matched cohort and sibling cohort study” by Deng et al**

We sincerely appreciate the editors’ and reviewers’ thoughtful and constructive comments. We have addressed each comment point by point in this response letter and revised the manuscript accordingly. We believe these comments and corresponding revisions have substantially strengthened our work. Apart from the revisions based on reviewers’ comments, we received new data during the revision process and therefore have extended the follow up from 2022 to 2024 in this version. We believe that the longer follow-up period and the increased number of study participants have further improved the manuscript.

A key reviewer concern is the definition of human papillomavirus (HPV) infection, as we used not only HPV testing but also cervical cytology as a proxy for HPV status. We would like to clarify first that, although cytology has lower sensitivity than HPV testing, it has still demonstrated high sensitivity for detecting cervical precancers and high concordance with HPV testing results in a Swedish context [Refs 1,2 in this letter]. Also, the lower sensitivity of cytology may increase false negatives, which will only attenuate the association between HPV infection and cardiovascular diseases (CVDs) in our study. This composite definition was adopted because HPV testing in Sweden has only been gradually implemented since 2015 and first achieved nationwide coverage in 2024. Therefore, using both cytology and HPV testing allowed for longer follow-up and improved statistical power, which are important to our research question. However, we acknowledge limitations of this approach and performed several subgroup and sensitivity analyses to ensure the results are robust. We have also discussed the limitations extensively.

Additionally, the attenuation of the CVD mortality association in previous sibling-matched analyses compared to population-matched analyses is also a key reviewer concern. In this revised version, notably, with an additional two years of follow-up, we now have sufficient statistical power for the sibling-matched analyses. The fully adjusted hazard ratios (HRs) and 95% confidence intervals (CIs) were 1.25 (95% CI: 1.16-1.34) for the population-matched analysis, with an identical point risk estimate (HR: 1.25) for the sibling-matched analysis albeit with a wider CI due to smaller numbers (95% CI: 1.01-1.53). Previously, the corresponding HRs were 1.27 (95% CI: 1.18-1.37) and 1.13 (95% CI: 0.90-1.40), respectively. Finally, we acknowledge the risk of reverse causation. To mitigate this concern, we conducted additional sensitivity analyses as requested by reviewers and expanded the Discussion, where all results suggested that reverse causation is unlikely to substantially influence the findings.

# ***Reviewer #1:*** *This is a cohort study that examined the associations of HPV infection with incident CVD and CVD-related mortality using the Swedish national register data. The study appears well conducted and the reporting was detailed. I have several comments:*

## *1. Can the authors please also report risk differences in the abstract.*

**Response:** Yes. Absolute rate differences have been added to both the abstract and the main text.

## *2. Please include the adjusted variables in the abstract's methods.*

**Response:** We have now listed the covariates in the Methods section of the abstract.

## *3. Line 100, 'source women' seems a weird term. Perhaps 'study population'?*

**Response:** Thanks for the comment. “Source women” has been replaced by “Study population” in this revised version.

## *4. Please specify why obesity, type 2 diabetes (T2D), hyperlipidaemia, COPD, cancers other than cervical cancer, mental disorders, maternal history of CIN3+, and parental history of CVD were selected as covariate and statue their hypothesised causal role.*

**Response:** Thank you for the suggestion. These factors were selected based on prior studies indicating their potential to confound the association between human papillomavirus (HPV) and cardiovascular diseases (CVDs) [Refs 6-8, 24 in the main text]. The rationale has now been incorporated into the Covariates section of the revised manuscript. In addition, a directed acyclic graph (Figure S1) has been added to illustrate their hypothesized roles.

## *5. For smoking nulliparity or missing were grouped together (Table 1) can they be separated? Also why only smoking during pregnancy was included not smoking in general?*

**Response:** Yes, we have reported nulliparity and missing categories separately for smoking during pregnancy. The reason that only smoking during pregnancy was presented is that smoking information in our dataset was obtained from the Medical Birth Registry, which collected data exclusively from women who presented in routine pregnancy visits, and data on smoking from the general population were unfortunately not available. We acknowledge this as a limitation of the study and have added it to the Discussion section of the revised version.

## *6. Can the authors provide more details of the flexible parametric models - as far as I know it is a group of models. Which models were used specifically and why?*

**Response:** The flexible parametric survival models were specified on the log cumulative hazard scale with natural splines for log(time) using four degrees of freedom. In the population-matched cohort, to account for the matching with replacement by year of birth, the models were adjusted for year of birth using natural splines with three degrees of freedom with robust variance estimation with individual-based clustering using the sandwich estimator. In the sibling cohort, to account for the sibling pairs being clustered, the flexible parametric models used a gamma frailty [Ref 3 in this letter]. We have now added these descriptions to the Statistical analysis section of the revised version.

## *7. Since the authors identified a time varying association between HPV test positive and CVD, I wondered if they also consider using the self-controlled case series to triangulate findings? They could either look at nonfatal events specifically or used the modified version for fatal and nonfatal events. One reason I asked for this method is because the HR is much stronger when HPV test positive was initially found. In fact many outcomes were non-significant after the first year of follow-up. I wondered there is any possibly of reverse causation and SCCS method (with before test positive period as a sensitivity exposure period) could help answer that question.*

**Response:** We thank the reviewer for this insightful suggestion. We agree that self-controlled case series can be helpful for assessing possible reverse causation. However, we did not consider it optimal in our setting for several reasons. First, the date of an HPV-positive test is not the true date of infection, as HPV may have been acquired years earlier and may persist before detection. In addition, any association between HPV and CVD is likely to be long-term, biologically gradual, and age-dependent rather than an acute post-exposure effect, making it difficult to define a meaningful self-controlled case series risk window. Moreover, our primary outcome included both fatal and nonfatal CVD events, whereas self-controlled case series is less straightforward for such outcomes. We have therefore not added this analysis, but we have clarified in the Discussion that the excess risk observed during the first year should be interpreted cautiously. Additionally, in response to Reviewer 3’s Comment 2 also related to reverse causation, we included sensitivity analyses by excluding outcome events that occurred within the first three months. The associations (Tables S7 and S10) remained unchanged, indicating a minimal impact of reverse causation if any.

## *8. The authors speculate the psychological stress due to an HPV test positive was a reason of the increased CVD risk in the first year of follow-up, which appeared the only possible reason for the time varying association. However, i wondered if the authors could provide some statistics to support this speculation, even basic descriptive statistics of proportion with anxiety in the exposed and unexposed group by year. This will add confidence to the potential causality of the findings.*

**Response:** Thanks for your comment. We have now added the Table S12, which presents the occurrence of new mental disorders after the follow-up start among women without prior mental disorders, stratified by follow-up year, within the population-matched cohorts. We observed that the number and proportion of women with incident mental disorders were highest in the first 1-2 follow-up years, gradually decreasing thereafter. These findings partially support our hypothesis that psychological stress after a positive HPV test may contribute to the elevated CVD risk in the early follow-up period. We also added this point in the Discussion of this revised version.

# ***Reviewer #2:*** *Thank you very much for this opportunity to review the manuscript, "Human papillomavirus infection and risk of cardiovascular disease: a population-based matched cohort and sibling cohort study. This is a very well-conducted study, and its findings have important implications. I have a few comments/questions.*

*Major comments*

## *1) The authors state in the abstract that "given the small absolute risk difference, women should not be excessively concerned." The authors also state that in the discussion. This may be true; however, they did not estimate risk difference or population-attributable fraction (PAF) for HPV infection in relation to major cardiovascular events. Therefore, as this inference extends beyond what the study's findings can support, I would recommend refraining from making this statement. Alternatively, the authors can estimate PAF and state whether PAF for HPV infection is relatively small or not.*

Response: Thank you for the comment. Absolute rate differences and population-attributable fractions have been added in this revised version, indicating that the absolute differences were small.

## *2) HPV vaccination is available during the time frame for this study. Would HPV vaccination reduce the risk of CVD? I think it would be very important to evaluate that. If that is beyond the scope of the study, it would be still important to account for that potential confounding factor.*

**Response:** Thank you. We have now included human papillomavirus (HPV) vaccination as a covariate in the adjusted model 2. Overall, the results and conclusions remain unchanged after this addition. Moreover, we have conducted subgroup analyses based on HPV vaccination (yes or no). We also agree that the association between HPV vaccination and cardiovascular diseases (CVDs) is an important topic; however, such an investigation would require longer follow-up, and we will definitely consider this in future studies.

## *3) The authors describe about four groups in the Method (page 11). However, I am not clear how they accounted for the four groups in the analysis. Group 4: Women who had only HPV testing during follow-up - That would be the ideal group of women to evaluate. As the authors discussed, cervical cytology (Pap smear) has a low sensitivity for detecting precancerous lesions and a cytology test is not an HPV test. The authors stated that HPV testing was recommended as the primary screening from 2015 in Sweden. Any changes in the assessment of exposure can affect the results. I think it would be important to look at the cohort (Group 4) based on those who were eligible for HPV testing from 2015 and see whether the findings are consistent in the sensitivity analysis.*

**Response:** Thank you for your comment. We would like to first clarify that, although cervical cytology has a lower sensitivity compared to HPV testing, it still demonstrates high sensitivity for detecting cervical precancerous lesions in a Swedish context. An earlier study in Sweden showed that the sensitivity of HPV testing and cytology for cervical intraepithelial neoplasia grade 3 or higher (CIN3+) was 97.0% and 91.6%, respectively [Ref 1 in this letter]. In addition, there is also a high concordance between cytological abnormalities and HPV testing [Ref 2 in this letter]. A study conducted in Sweden reported that approximately 76.7% of women with high-grade squamous intraepithelial lesions (HSIL) tested positive for HPV [Ref 2 in this letter]. Among HPV-positive HSIL samples, high-risk HPV genotypes accounted for 97.1% [Ref 2 in this letter]. Moreover, compared with HPV testing, the lower sensitivity of cervical cytology is more likely to yield a higher proportion of false-negative results, thereby only attenuating hazard ratios (HRs) for CVD in HPV+ individuals.

Furthermore, three out of the four HPV infection groups (Groups 2, 3, and 4) in our study utilized HPV testing results, not just Group 4, which included women who only had HPV testing but not cytology during follow-up. Conducting a subgroup analysis solely based on Group 4 may not be informative, as this group has a small sample size and short follow-up, which might lead to imprecise estimates.

The reason we used both cytology and HPV testing to define HPV infection in this study is that this approach allows for a longer follow-up period (since exposure/HPV infections) and identifies more individuals with the exposure, thereby increasing statistical power. However, we acknowledge that in Groups 2 and 3, the 30-day cutoff used to define HPV infection between cytology and HPV testing was arbitrary. In response, we have added two sensitivity analyses, using two different cutoffs (15 and 60 days) to define HPV infection. The results (Tables S7 and S10) were consistent with the main findings.

## *4) In the prior cohort study in Korean women, the authors investigated high-risk HPV infection (16, 18, and other genotypes) and the incidence of CVD (HR = 1.25, 95% CI: 1.03-1.52). High-risk HPV can induce the degradation of the tumor-suppressor protein p53, which has been linked to accelerated atherosclerosis. HR from the Korean study was slightly higher than that of your study in Sweden (based on cytology or HPV test) because they specifically evaluated high-risk HPV. Please consider discussing that.*

**Response:** Thank you for the suggestions. As the reviewer pointed out, our use of both cytology and HPV test for our exposure may partially explain the differences in HRs vs. Korean studies. Another possible reason for the differences is that the Korean studies relied on single HPV test results, which may have included persistent HPV infections acquired many years ago. In contrast, our study only included incident HPV infections during the follow-up period, and we excluded women with prior HPV infections (as well as CVD) to ensure the correct temporal relationship between exposure and outcome. As a result, the HRs for potential persistent HPV infections in Korean studies are likely to be higher than the HRs among individuals with incident HPV infections in our study. In Sweden, HPV screening has been rolled out gradually since 2015 and only reached nationwide coverage in 2024. We have incorporated the above explanations in the Discussion section.

# *Reviewer #3: This manuscript reports a large and well-conducted population-based cohort study examining the link between HPV infection and the risk of developing cardiovascular disease (CVD) and CVD-related death.*

*The methods are strong, the writing is clear, and the findings add valuable evidence to an area where research is still limited. The time-varying analyses and the extensive sensitivity and subgroup analyses make the results more reliable.*

*Overall, the manuscript appears suitable for publication after minor revisions.*

*Comments*

## *1. The definition of HPV infection is based on either (1) abnormal cytology or (2) a positive HPV test. Since cytology has lower sensitivity and does not always reflect true HPV infection, this could lead to non-differential misclassification and bias the results toward the null. Although the authors mention this, it would be helpful to explain it more clearly or quantify it. If possible, please provide descriptive information or a supplemental table comparing the baseline characteristics of the four exposure groups.*

**Response:** Thank you for the comment. We have now added two supplemental tables (Tables S3 and S4) to present the characteristics of the study population across the four exposure groups. We would like to first clarify that, although cervical cytology has a lower sensitivity compared to HPV testing, it still demonstrates high sensitivity for detecting cervical precancerous lesions in a Swedish context. An earlier study in Sweden showed that the sensitivity of HPV testing and cytology for cervical intraepithelial neoplasia grade 3 or higher (CIN3+) was 97.0% and 91.6%, respectively [Ref 1 in this letter]. In addition, there is also a high concordance between cytological abnormalities and HPV testing [Ref 2 in this letter]. A study conducted in Sweden reported that approximately 76.7% of women with high-grade squamous intraepithelial lesions (HSIL) tested positive for HPV [Ref 2 in this letter]. Among HPV-positive HSIL samples, high-risk HPV genotypes accounted for 97.1% [Ref 2 in this letter]. Moreover, compared with HPV testing, the lower sensitivity of cervical cytology is more likely to yield a higher proportion of false-negative results, thereby only attenuating the hazard ratios (HRs) for HPV infection and cardiovascular diseases (CVDs).

The reason we used both cytology and HPV testing to define HPV infection in this study is that this approach allows for a longer follow-up period (since exposure/HPV infections) and identifies more individuals with the exposure, thereby increasing statistical power. However, we acknowledge that in Groups 2 and 3, the 30-day cutoff used to define HPV infection between cytology and HPV testing was arbitrary. In response, we have added two sensitivity analyses, using two different cutoffs (15 and 60 days) to define HPV infection. The results (Tables S7 and S10) were consistent with the main findings.

## *2. The authors report that CVD incidence and mortality are highest in the first year and then decrease. To address the possibility of reverse causation, the manuscript would benefit from an additional sensitivity analysis that excludes events occurring in the first 3 months.*

**Response:** Thank you. We conducted a sensitivity analysis by excluding CVD events that occurred within the first three months. The results (Tables S7 and S10) showed that the associations remained consistent with the main findings, indicating a minimal impact, if any, from reverse causation.

## *3. The sibling comparison is an important strength of the study, but the reduced association with mortality raises questions. Although the limited number of deaths among siblings may weaken the statistical power, it would be helpful for the authors to discuss more clearly how shared family factors (such as socioeconomic status or lifestyle behaviors) could influence mortality differently from CVD incidence.*

**Response:** It is true that shared family factors, such as genetic predispositions, early-life exposures, or common lifestyle behaviors, may have a more significant effect on the risk of CVD-related death than on CVD incidence, which could partially explain the attenuation observed in the sibling-matched analysis. On the other hand, we would like to highlight an update made to the study, where the follow-up period was extended by two additional years, changing the study end year from 2022 to 2024. The updated results increased the statistical power of the sibling-matched analysis. The fully adjusted HRs and 95% CIs were 1.25 (95% CI: 1.16-1.34) for the population-matched analysis and 1.25 (95% CI: 1.01-1.53) for the sibling-matched analysis. Previously, the corresponding HRs were 1.27 (95% CI: 1.18-1.37) and 1.13 (95% CI: 0.90-1.40), respectively.

## *4. With small revisions that clarify the exposure definition, expand the interpretation of the early peak in risk, and refine the discussion of potential mechanisms, the manuscript will be even stronger and ready for publication.*

**Response:** We want to thank the peer reviewer for his/her positive and constructive feedback.

# *Reviewer #4: Thank you for the opportunity to review this manuscript. The study uses large national registries in Sweden to investigate the relationship between HPV infection and cardiovascular disease. The research question is important and the dataset provides strong potential for meaningful insights. The work is valuable, but several concerns need to be addressed before the manuscript is suitable for publication.*

*Major comments*

## *1. The diagnostic definition of HPV infection needs to be reconsidered. Combining cytology-based detection with HPV-testing-based detection introduces substantial heterogeneity in exposure classification. Although the authors acknowledge cytology's lower sensitivity (Lines 343-348), the issue is more fundamental. Women in Group 1 (cytology only) likely represent a different population with different healthcare-seeking behaviors compared with Groups 2-4. In addition, the 30-day threshold used for Group 2 appears arbitrary and lacks justification or citation.*

**Response:** Thanks for the comment. We would like to first clarify that, although cervical cytology has a lower sensitivity compared to HPV testing, it still demonstrates high sensitivity for detecting cervical precancerous lesions in a Swedish context. An earlier study in Sweden showed that the sensitivity of HPV testing and cytology for cervical intraepithelial neoplasia grade 3 or higher (CIN3+) was 97.0% and 91.6%, respectively [Ref 1 in this letter]. In addition, there is also a high concordance between cytological abnormalities and HPV testing [Ref 2 in this letter]. A study conducted in Sweden reported that approximately 76.7% of women with high-grade squamous intraepithelial lesions (HSIL) tested positive for HPV [Ref 2 in this letter]. Among HPV-positive HSIL samples, high-risk HPV genotypes accounted for 97.1% [Ref 2 in this letter]. Moreover, compared with HPV testing, the lower sensitivity of cervical cytology is more likely to yield a higher proportion of false-negative results, thereby only attenuating hazard ratios (HRs) for HPV infection and cardiovascular diseases (CVDs). In addition, we have also added new supplemental tables (Tables S3 and S4) to present the characteristics of the study population across the four exposure groups.

The reason we used both cytology and HPV testing to define HPV infection in this study is that this approach allows for a longer follow-up period (since exposure/HPV infections) and identifies more individuals with the exposure, thereby increasing statistical power. However, we acknowledge that in Groups 2 and 3, the 30-day cutoff used to define HPV infection between cytology and HPV testing was arbitrary. In response, we have added two sensitivity analyses, using two different cutoffs (15 and 60 days) to define HPV infection. The results (Tables S7 and S10) were consistent with the main findings.

## *2. The markedly elevated risk observed in the first year after HPV detection (HR = 1.50 for incident CVD; HR = 2.92 for mortality) raises important concerns regarding reverse causation and detection bias. While the authors mention psychological stress and surveillance bias (Lines 289-301), the issue is understated. Women experiencing early cardiovascular symptoms may seek medical attention more frequently, increasing the likelihood of opportunistic cervical screening and HPV detection.*

**Response:** We agree that reverse causation and detection bias are possible. We have discussed it as a limitation. Furthermore, in response to Reviewer 3’s Comment 2, which is also related to reverse causation, we conducted an additional sensitivity analysis in this version by excluding events that occurred within the first three months. The results (Tables S7 and S10) indicated that the associations remained unchanged.

In addition, in response to Reviewer 1’s Comment 8, to support our hypothesis that psychological stress resulting from a positive cervical screening result may contribute to the elevated CVD risk in the early follow-up period, we presented the occurrence of new mental disorders after the follow-up start among women without prior mental disorders, stratified by follow-up year, within the population-matched cohorts (Table S12). We observed that the number and proportion of women with new mental disorders were highest in the first 1-2 follow-up years, gradually decreasing thereafter.

## *3. The attenuation of the CVD mortality association in the sibling comparison analysis (HR = 1.13, 95% CI: 0.90-1.40) warrants a more thoughtful interpretation. The authors suggest this may be due to limited statistical power, but the point estimate shifts meaningfully from 1.27 to 1.13, and the confidence interval includes the null. This pattern suggests that familial confounding may contribute to the observed association. The conclusion recommending clinicians remain "alert" to CVD risk (Lines 367-368) appears overstated in light of these findings.*

**Response:** Thank you. We noted that “alert” may not be an appropriate word for the conclusion and have attenuated our statements throughout the manuscript. We would also like to highlight an update made to the study, where the follow-up period was extended by two additional years, changing the study end year from 2022 to 2024. The updated results showed that we now may have sufficient power for the sibling-matched analysis. The fully adjusted HRs and 95% CIs were 1.25 (95% CI: 1.16-1.34) for the population-matched analysis and 1.25 (95% CI: 1.01-1.53) for the sibling-matched analysis. Previously, the corresponding HRs were 1.27 (95% CI: 1.18-1.37) and 1.13 (95% CI: 0.90-1.40), respectively.

## *4. The mechanistic section (Lines 317-329) is too limited and relies on older or small-scale studies. It omits recent comprehensive reviews, including Saha et al. (*[*https://eur01.safelinks.protection.outlook.com/?url=https%3A%2F%2Fdoi.org%2F10.1007%2Fs11010-025-05236-9&data=05%7C02%7Cyunyang.deng%40ki.se%7Cfeb4777e108f40d05b9a08de3d697b56%7Cbff7eef1cf4b4f32be3da1dda043c05d%7C0%7C0%7C639015720636780205%7CUnknown%7CTWFpbGZsb3d8eyJFbXB0eU1hcGkiOnRydWUsIlYiOiIwLjAuMDAwMCIsIlAiOiJXaW4zMiIsIkFOIjoiTWFpbCIsIldUIjoyfQ%3D%3D%7C0%7C%7C%7C&sdata=5W4OjRytndT6A6kvYhJCN%2Fb6P3NfCBosjmSCSFWNmpg%3D&reserved=0*](https://doi.org/10.1007/s11010-025-05236-9)*), which detail key pathways such as E6/E7-mediated endothelial dysfunction, chronic inflammation, oxidative stress, altered lipid metabolism, and distinctions between persistent and transient HPV infection. Including these contemporary insights would strengthen the biological rationale.*

**Response:** Thank you for this useful resource. We have discussed the mechanisms and included the mentioned reference.

*Minor comments*

## *1. In Table 1, the exposed group shows a higher rate of nulliparity (54.45% vs 41.39%), which is surprising given that HPV is sexually transmitted. This warrants comment and may indicate selection bias related to differences in screening participation.*

**Response:** Thank you for the comment. We agree that sexual activity is an important factor for HPV infection. However, unfortunately, we do not have data on sexual behaviors such as age at first intercourse, number of sexual partners, or condom use. In this revised version, by extending the follow-up period by two additional years, the difference in nulliparity is smaller in this updated version (57.17% vs. 52.53%). Moreover, all participants in this study had at least one cervical cancer screening result during follow-up to determine HPV infection status, minimizing the likelihood of selection bias related to differences in screening participation.

## *2. CVD subtypes (Lines 215-217, 244-251): The heterogeneity in associations across CVD subtypes is not adequately addressed. It is unclear why HPV infection would be related to arrhythmias but not consistently to ischemic heart disease. This pattern is more compatible with detection bias than with a biological mechanism involving atherosclerosis.*

**Response:** Indeed, heterogeneity across CVD subtypes is a concern in this study. We speculate that one potential reason is that only the first CVD record during follow-up was extracted for each participant, which may preferentially capture less severe CVD cases. Furthermore, since our study population is relatively young at index, diagnosed CVDs are more likely to be less severe, as more severe CVDs typically occur in middle-aged or older individuals. Also, some CVD subtypes may be associated with mental disorders [Ref 4 in this letter], which may act as a mediator in the association between HPV infection and CVD in our study. We have included these points in the Discussion.

## *3. In the Discussion (Lines 275-278), the claim that "women should not be unduly worried" contradicts the subsequent suggestion that clinicians should be "alert" to CVD risk. The authors should provide absolute risk differences in addition to relative risks in both the abstract and discussion to contextualize the findings.*

**Response:** Thank you for the comment. Absolute rate differences and population-attributable fractions have been added in this revised version. Moreover, we have attenuated our statements throughout the manuscript.

## *4. In Lines 351-356, the limitation regarding HPV clearance and reinfection is understated. Persistent HPV infection represents a fundamentally different biological and clinical state than transient infection, yet the study cannot distinguish between the two. This is more than a "dynamic nature" issue; it is a meaningful exposure misclassification problem.*

**Response:** Thank you for the comment. We have incorporated this point into the limitation part in the current version.

## *5. The study does not address HPV infection or post-infection outcomes in males, which limits broader interpretation of the findings.*

**Response:** Thanks for the comment. We have added this as a limitation.

# **Reference:**

1. Hortlund M, Mühr LSA, Lagheden C, Hjerpe A, Dillner J. Audit of laboratory sensitivity of human papillomavirus and cytology testing in a cervical screening program. Int J Cancer. 2021;149(12):2083-2090.
2. García FR, Norenhag J, Edfeldt G, Cheng L, Hugerth LW, Pennhag AAL, et al. Prevalence of the human papillomavirus (HPV) types among cervical dysplasia women attending a gynaecological clinic in Sweden. BJC Rep. 2023;1(1):11.
3. Liu XR, Pawitan Y, Clements MS. Generalized survival models for correlated time-to-event data. Stat Med. 2017;36(29):4743-62.
4. Thrall G, Lip GY, Carroll D, Lane D. Depression, anxiety, and quality of life in patients with atrial fibrillation. Chest. 2007;132(4):1259-64.
