# Supplementary material for: Human papillomavirus infection and risk of cardiovascular disease: A population-based matched cohort study with sibling analysis
Source: PLoS Med. 2026 Sep 1;23(9):e1005222. doi: 10.1371/journal.pmed.1005222 (PMC13533340; doi:10.1371/journal.pmed.1005222)
Supplement: Supplementary file 16 [file pmed.1005222.s017.docx]

**Response to comments on manuscript PMEDICINE-D-26-01654R1 entitled “Human papillomavirus infection and risk of cardiovascular disease: a population-based matched cohort and sibling cohort study” by Deng et al**

We sincerely thank the editors and reviewers for their thoughtful and constructive comments. We have addressed each comment individually in this response letter and revised the manuscript accordingly. We believe that these comments and the corresponding revisions have significantly improved the manuscript. In addition, we reviewed the reference list to ensure its completeness and accuracy. No changes were made to the reference list during the revision.

# ***Requests from Editors:***

*GENERAL EDITORIAL REQUESTS*

## * At this stage, we ask that you include a short, non-technical Author Summary of your research to make findings accessible to a wide audience that includes both scientists and non-scientists. The Author Summary should immediately follow the Abstract in your revised manuscript. This text is subject to editorial change and should be distinct from the scientific abstract. Ideally each sub-heading should contain 2-3 single sentence, concise bullet points containing the most salient points from your study. In the final bullet point of ‘What Do These Findings Mean?’ Please include the main limitations of the study in non-technical language. Please see our author guidelines for more information.

**Response:** The Author Summary has been added immediately after the Abstract in the revised manuscript.

## * Please confirm that your title complies with PLOS Medicine's style. Your title must be nondeclarative and not a question. It should begin with main concept if possible. "Effect of" should be used only if causality can be inferred, i.e., for an RCT. Please place the study design ("A randomized controlled trial," "A retrospective study," "A modelling study," etc.) in the subtitle (ie, after a colon). We suggest the following title: “Human papillomavirus infection and risk of cardiovascular disease: A population-based cohort study with sibling analysis”.

**Response:** Based on your suggestion, we have revised the title to “Human papillomavirus infection and risk of cardiovascular disease: A population-based matched cohort study with sibling analysis”.

## * Please ensure that all abbreviations are defined at first use throughout the text.

**Response:** We have carefully reviewed the manuscript and ensured that all abbreviations are defined at their first occurrence.

## * Please confirm that all numbers presented in the abstract are present and identical to numbers presented in the main manuscript text.

**Response:** We have carefully reviewed the manuscript and confirmed that all numbers reported in the Abstract are consistent with those presented in the main text.

## * Please disclose any use of AI tools and technologies to the study or article content at the end of the Methods. Please specify the name(s) and version(s) of any tool(s) used and a description of their use, including how the AI-assisted or generated content was evaluated by authors, and a clear statement of which aspects of the study, article contents, data, or supporting files were affected/generated by AI tool usage. Please also include a statement specifying who retains responsibility for the final manuscript. See our guidelines for more information.

**Response:** The disclosure regarding the use of AI tools has been added at the section of Methods - Disclosure of the use of artificial intelligence tools.

## * Statistical reporting: Please revise throughout the manuscript, including tables and figures.

*- Please report statistical information as follows to improve clarity for the reader ""22% (95% CI [13,28]; p</=)"".*

*- Please separate upper and lower bounds with commas instead of hyphens as the latter can be confused with reporting of negative values.*

*- Please repeat statistical definitions (HR, CI etc.) for each set of parentheses.*

**Response:** We have revised the manuscript and related materials to ensure compliance with the statistical reporting requirements.

## * Please include your Ethics statement in the Methods section of your manuscript.

**Response:** The Ethics statement has been added in the section of Methods - Ethical statement.

*ABSTRACT*

## * Please confirm that your abstract complies with our requirements, including format (three sections: Background, Methods and Findings, and Conclusions) and providing all the information relevant to this study type.

**Response:** The Abstract has been revised to comply with the required format, specify the study type, and fulfill the other Abstract requirements.

## * Please include the numerical results for the sibling analysis in the abstract.

**Response:** The numerical results from the sibling analyses have been added to the Methods and Findings section of the Abstract.

## * In the last sentence of the Abstract Methods and Findings section, please describe the main limitation(s) of the study's methodology.

**Response:** The main limitations of the study’s methodology have been added at the end of the Methods and Findings section of the Abstract.

*FUNDING STATEMENT*

## * The funding statement should include: specific grant numbers, initials of authors who received each award, URLs to sponsors’ websites. Also, please state whether any sponsors or funders (other than the named authors) played any role in study design, data collection and analysis, the decision to publish, or preparation of the manuscript. If they had no role in the research, include this sentence: “The funders had no role in study design, data collection and analysis, decision to publish, or preparation of the manuscript.”

## * Please include URLs to the funders’ websites.

**Response:** We have updated the funding statement to include the funder names, specific grant numbers (where applicable), initials of the authors who received each award, and URLs to the funders’ websites. The sentence of the funders’ role has also been revised.

*DATA AVAILABILITY*

## * PLOS Medicine requires that the de-identified data underlying the specific results in a published article be made available, without restrictions on access, in a public repository or as Supporting Information at the time of article publication, provided it is legal and ethical to do so.

## * The Data Availability Statement (DAS) requires revision. For each data source used in your study:

*a) If the data are freely or publicly available, note this and state the location of the data: within the paper, in Supporting Information files, or in a public repository (include the DOI or accession number).*

*b) If the data are owned by a third party but freely available upon request, please note this and state the owner of the data set and contact information for data requests (web or email address). Note that a study author cannot be the contact person for the data.*

*c) If the data are not freely available, please describe briefly the ethical, legal, or contractual restriction that prevents you from sharing it. Please also include an appropriate contact (web or email address) for inquiries (again, this cannot be a study author).*

**Response:** Our study falls under situation c), where the data are not publicly available. Accordingly, the Data Availability Statement has been revised to include appropriate non-author contacts for data access inquiries.

*FIGURES AND TABLES*

## * Please provide titles and legends for all figures and tables (including those in Supporting Information files). Please define all acronyms used in each figure or table in its corresponding legend.  Currently the titles are quite long and detailed, and there are no legends. Please amend.

**Response:** In the revised version, titles and legends (if applicable) for all figures and tables, including those in the Supporting Information, have been added to the manuscript. In addition, the titles of several figures and tables have been shortened.

## * Please consider if moving some of the figures/tables shared in the supplementary information into the main text would aid the reader. Specifically, we would suggest to move figure S5 to the main manuscript.

**Response:** In the previous submission, there was only one supplementary figure (Figure S1). In this revised version, this figure has been moved to the main text and is now presented as Figure 5.

*OBSERVATIONAL, COHORT, CROSS-SECTIONAL, AND CASE CONTROL STUDIES*

## * Please ensure that the study is reported according to the STROBE guideline, and include the completed STROBE checklist as Supporting Information.  Please add the following statement, or similar, to the Methods: ""This study is reported as per the Strengthening the Reporting of Observational Studies in Epidemiology (STROBE) guideline (S1 Checklist)."" When completing the checklist, please use section and paragraph numbers, rather than page numbers.

**Response:** The STROBE checklist has been added and uploaded as a Supporting Information file (S2 Checklist), and relevant statement has been added in the Methods section.

## * Did your study have a prospective protocol or analysis plan? Please state this (either way) early in the Methods section.

*a) If a prospective analysis plan (from your funding proposal, IRB or other ethics committee submission, study protocol, or other planning document written before analyzing the data) was used in designing the study, please include the relevant prospectively written document with your revised manuscript as a Supporting Information file to be published alongside your study, and cite it in the Methods section. A legend for this file should be included at the end of your manuscript.*

*b) If no such document exists, please make sure that the Methods section transparently describes when analyses were planned, and when/why any data-driven changes to analyses took place.*

*c) In either case, changes in the analysis-- including those made in response to peer review comments-- should be identified as such in the Methods section of the paper, with rationale.*

**Response:** We have included a prospective research plan as Supporting Information (S1 Research Plan) and specified this in the Methods section - Data source section of the revised manuscript. The changes made to the analyses during peer review, together with their corresponding rationales, have also been documented in the S1 Research Plan.

# ***Journal Requirements:***

## 1) Please provide separate figure files in .tif or .eps format. For more information about figure files please see our guidelines.

**Response:** We have provided all figures in TIFF format now.

## 2) We note that you have uploaded Table 1 as Manuscript file. Table should not be uploaded as individual files. Please remove this file and include the Table in your manuscript file as editable, cell-based objects. For more information about how to format tables, see our guidelines.

**Response:** Table 1 has been moved to the end of the manuscript in the revised version.

## 3) Please note that the contact for accessing your data cannot be an author on the manuscript. Please therefore replace the current contact information with a third party contact, such as an institutional data access committee.

**Response:** The Data Availability Statement has been revised to include appropriate non-author contacts for data access inquiries.

# ***Reviewer #1:*** *Thank you very much for addressing my questions. I do not have any further comments.*

**Response:** Thanks for your positive comment.

# ***Reviewer #2:*** *Thank you for responding to my comments. I appreciate, particularly, on showing primary data in new mental health conditions following HPV test positive, which was convincing to the explanation.*

# *I have only one minor suggestion - it isn't immediately clear how 'comorbidity' (as in Figure S1 the DAG) could influence HPV test positive. Could you please expand that in the Methods / Supplementary materials using hyperlipidaemia as an example?*

**Response:** Thanks for the comment. We have expanded this point in the Methods - Covariates section of the revised manuscript using obesity, type 2 diabetes, and hyperlipidemia as examples.

# ***Reviewer #3:*** *Thank you for the revised manuscript and the detailed responses to the reviewers' comments. The authors have addressed most of the concerns raised, and the manuscript has improved considerably. However, one point still requires attention before the manuscript is ready for publication.*

# *In the author response, the authors noted that shared family factors—such as genetic background, early-life experiences, and similar lifestyle habits—may play a stronger role in CVD-related mortality than in CVD incidence, and that this may partly explain why the association was weaker in the sibling-matched analysis. However, this point is not discussed in the revised manuscript. We suggest that the authors include this explanation in the Discussion section.*

**Response:** Thank you for this important comment. We would like to clarify that, after extending the follow-up period by two additional years, the updated results showed comparable estimates between population-matched and sibling-matched analyses for both outcomes. Specifically, for incident CVD, the fully adjusted HRs (95% CIs) were 1.07 (1.05, 1.08) in the population-matched analysis and 1.05 (1.01, 1.08) in the sibling-matched analysis. For CVD-related mortality, the corresponding estimates were 1.25 (1.16, 1.34) and 1.25 (1.01, 1.53), respectively.

Nevertheless, we agree with the reviewer that shared familial factors may influence the observed associations and could have different impacts on CVD incidence and mortality. We have therefore added a clarification in the Discussion section to acknowledge the potential role of shared familial factors as a possible source of residual confounding in sibling comparisons (Discussion - Paragraph 6).

# ***Reviewer #4:*** *The authors have adequately addressed the comments raised during the previous round of review. They have added analyses of absolute risk differences and population-attributable fractions, incorporated HPV vaccination into the adjusted models, performed additional subgroup and sensitivity analyses, and expanded the Discussion to clarify differences from previous studies. These revisions have strengthened the manuscript and improved its interpretation.*

# *The study is well designed, the analyses are rigorous, and the findings provide important evidence regarding the association between HPV infection and cardiovascular disease. I have no further substantive comments and recommend acceptance of the manuscript in its current form.*

**Response:** Thanks for your positive comment.
