# Supplementary material for: Human papillomavirus infection and risk of cardiovascular disease: A population-based matched cohort study with sibling analysis
Source: PLoS Med. 2026 Sep 1;23(9):e1005222. doi: 10.1371/journal.pmed.1005222 (PMC13533340; doi:10.1371/journal.pmed.1005222)
Supplement: Supplementary file 17 [file pmed.1005222.s018.docx]

**Response to comments on manuscript PMEDICINE-D-26-01654R2 entitled “Human papillomavirus infection and risk of cardiovascular disease: A population-based matched cohort study with sibling analysis” by Deng et al**

We sincerely thank the editors for the additional thoughtful comments. We have addressed each comment individually in this response letter and revised the manuscript accordingly. We believe that these comments and the corresponding revisions have strengthened the manuscript.

# ***Requests from Editors:***

## *1) Statistical reporting: Thank you for amending your statistical reporting. However, we note that in the tables you still use round brackets rather than square ones. Please amend, so that the text, the figures and tables all follow our statistical guidelines*:

*- Please report statistical information as follows to improve clarity for the reader "22% (95% CI [13,28]; p</=)".*

*- Please separate upper and lower bounds with commas instead of hyphens as the latter can be confused with reporting of negative values.*

*- Please repeat statistical definitions (HR, CI etc.) for each set of parentheses.*

**Response:** Thanks for your kind reminder, and we apologize for this oversight. We have revised the text, tables, and figures to ensure compliance with the statistical reporting requirements.

## 2) Competing interests: Please include a statement “The other authors declare that no competing interests exist.”

**Response:** We have added the sentence to the Disclosure of interest section.

## 3) Data availability: thank you for providing contact details from someone that is not the study author. However, please provide more detail on the requirement for researchers to gain access to the data.

**Response:** We have provided additional details on how to access the data in the Data availability section.

## 4) Figure 4: Please include the variables that were adjusted for in the legend.

**Response:** We have added information regarding the adjusted variables to the legend of Figure 4.

## 5) This is a mistake on my part, please accept my apologies. In my previous decision, I've asked "Please consider if moving some of the figures/tables shared in the supplementary information into the main text would aid the reader. Specifically, we would suggest to move figure S5 to the main manuscript." However, I did mean Table S5.

*a) Please consider whether moving TABLE S5 into the main manuscript would aid the reader.*

*b) Thank you for moving figure S1 to the main text, due to my erroneous request. I will leave it up to you whether you want to leave this figure in the main text, or move it back to the supplements.*

**Response:** Thank you for the clarification. We have moved Table S5 to the main manuscript as Table 2. Meanwhile, we would like to move Figure S1 back to the Supplements.
