# Supplementary material for: Human papillomavirus infection and risk of cardiovascular disease: A population-based matched cohort study with sibling analysis
Source: PLoS Med. 2026 Sep 1;23(9):e1005222. doi: 10.1371/journal.pmed.1005222 (PMC13533340; doi:10.1371/journal.pmed.1005222)
Supplement: Peer review history

# Author comment: Author response to Decision Letter 0

**DOI:** 10.1371/journal.pmed.1005222.r001

15 May 2026

Attachment Submitted filename: Response letter_4.0_260515_clean.docx

# Editor report: Decision Letter 0

**DOI:** 10.1371/journal.pmed.1005222.r002

**Authors:** Suzanne De Bruijn

22 May 2026

Dear Dr Deng,

Thank you for submitting your manuscript entitled "Human papillomavirus infection and risk of cardiovascular disease: a population-based matched cohort and sibling cohort study" for consideration by PLOS Medicine.

Your manuscript has now been evaluated by the PLOS Medicine editorial staff, and I am writing to let you know that we would like to send your submission out for external peer review.

However, before we can send your manuscript to reviewers, we need you to complete your submission by providing the metadata that is required for full assessment. To this end, please login to Editorial Manager where you will find the paper in the 'Submissions Needing Revisions' folder on your homepage. Please click 'Revise Submission' from the Action Links and complete all additional questions in the submission questionnaire.

For clinical studies, please upload a copy of your trial study protocol as a supporting information file. The study protocol should be the version submitted for approval to the institutional review board or ethics committee, should include any amendments to the study protocol, as well as the date of their approval by the institutional review or ethics committee. Please also detail any deviations from the study protocol in the Methods section of your manuscript. The editors will consider the protocol and study conduct prior to a final decision for external review.

Please re-submit your manuscript within two working days, i.e. by May 26 2026 11:59PM.

Login to Editorial Manager here: https://www.editorialmanager.com/pmedicine

Once your full submission is complete, your paper will undergo a series of checks in preparation for peer review. Once your manuscript has passed all checks it will be sent out for review.

Feel free to email us at plosmedicine@plos.org if you have any queries relating to your submission.

Kind regards,

Suzanne De Bruijn, PhD

Associate Editor

PLOS Medicine

# Author comment: Author response to Decision Letter 1

**DOI:** 10.1371/journal.pmed.1005222.r003

22 May 2026

Attachment Submitted filename: Response_letter_4.0_260515_clean_auresp_1.docx

# Peer review: Decision Letter 1

**DOI:** 10.1371/journal.pmed.1005222.r004

**Authors:** Suzanne De Bruijn

7 Jul 2026

Dear Dr. Deng,

Thank you very much for re-submitting your manuscript "Human papillomavirus infection and risk of cardiovascular disease: a population-based matched cohort and sibling cohort study" (PMEDICINE-D-26-01654R1) for review by PLOS Medicine.

I have discussed the paper with my colleagues and the academic editor and it was also seen again by 4 reviewers. The reviewers have only minor remaining concerns. I am pleased to say that provided the remaining reviewer concerns, as well as the editorial and production issues are dealt with we are planning to accept the paper for publication in the journal.

The remaining issues that need to be addressed are listed at the end of this email. Any accompanying reviewer attachments can be seen via the link below. Please take these into account before resubmitting your manuscript:

[LINK]

Please also address the editorial requests, which you can find at the bottom of this email. Please include these in your point-by-pont response.

***Please note while forming your response, if your article is accepted, you may have the opportunity to make the peer review history publicly available. The record will include editor decision letters (with reviews) and your responses to reviewer comments. If eligible, we will contact you to opt in or out.***

In revising the manuscript for further consideration here, please ensure you address the specific points made by each reviewer and the editors. In your rebuttal letter you should indicate your response to the reviewers' and editors' comments and the changes you have made in the manuscript. Please submit a clean version of the paper as the main article file. A version with changes marked must also be uploaded as a marked up manuscript file.

Please also check the guidelines for revised papers at http://journals.plos.org/plosmedicine/s/revising-your-manuscript for any that apply to your paper. If you haven't already, we ask that you provide a short, non-technical Author Summary of your research to make findings accessible to a wide audience that includes both scientists and non-scientists. The Author Summary should immediately follow the Abstract in your revised manuscript. This text is subject to editorial change and should be distinct from the scientific abstract.

In addition to these revisions, you may need to complete some formatting changes, which you will receive in a follow up email. A member of our team will be in touch with a set of requests shortly. If you do not receive a separate email within a few days, please assume that checks have been completed, and no additional changes are required.

We expect to receive your revised manuscript within 1 week. Please email us (plosmedicine@plos.org) if you have any questions or concerns.

Please review your reference list to ensure that it is complete and correct. If you have cited papers that have been retracted, please include the rationale for doing so in the manuscript text, or remove these references and replace them with relevant current references. Any changes to the reference list should be mentioned in the rebuttal letter that accompanies your revised manuscript.

Please note, when your manuscript is accepted, an uncorrected proof of your manuscript will be published online ahead of the final version, unless you've already opted out via the online submission form. If, for any reason, you do not want an earlier version of your manuscript published online or are unsure if you have already indicated as such, please let the journal staff know immediately at plosmedicine@plos.org.

If you have any questions in the meantime, please contact me or the journal staff on plosmedicine@plos.org.

We look forward to receiving the revised manuscript by Jul 14 2026 11:59PM.

Sincerely,

Suzanne De Bruijn, PhD

Associate Editor

PLOS Medicine

plosmedicine.org

------------------------------------------------------------

Requests from Editors:

GENERAL EDITORIAL REQUESTS

* At this stage, we ask that you include a short, non-technical Author Summary of your research to make findings accessible to a wide audience that includes both scientists and non-scientists. The Author Summary should immediately follow the Abstract in your revised manuscript. This text is subject to editorial change and should be distinct from the scientific abstract. Ideally each sub-heading should contain 2-3 single sentence, concise bullet points containing the most salient points from your study. In the final bullet point of ‘What Do These Findings Mean?’ Please include the main limitations of the study in non-technical language.

Please see our author guidelines for more information: https://journals.plos.org/plosmedicine/s/revising-your-manuscript#loc-author-summary."

* Please confirm that your title complies with PLOS Medicine's style. Your title must be nondeclarative and not a question. It should begin with main concept if possible. "Effect of" should be used only if causality can be inferred, i.e., for an RCT. Please place the study design ("A randomized controlled trial," "A retrospective study," "A modelling study," etc.) in the subtitle (ie, after a colon).

* We suggest the following title: “Human papillomavirus infection and risk of cardiovascular disease: A population-based cohort study with sibling analysis”.

* Please ensure that all abbreviations are defined at first use throughout the text.

* Please confirm that all numbers presented in the abstract are present and identical to numbers presented in the main manuscript text.

* Please disclose any use of AI tools and technologies to the study or article content at the end of the Methods. Please specify the name(s) and version(s) of any tool(s) used and a description of their use, including how the AI-assisted or generated content was evaluated by authors, and a clear statement of which aspects of the study, article contents, data, or supporting files were affected/generated by AI tool usage. Please also include a statement specifying who retains responsibility for the final manuscript. See our guidelines for more information: https://journals.plos.org/plosmedicine/s/ethical-publishing-practice#loc-artificial-intelligence-tools-and-technologies.

* Statistical reporting: Please revise throughout the manuscript, including tables and figures.

- Please report statistical information as follows to improve clarity for the reader ""22% (95% CI [13,28]; p</=)"".

- Please separate upper and lower bounds with commas instead of hyphens as the latter can be confused with reporting of negative values.

- Please repeat statistical definitions (HR, CI etc.) for each set of parentheses.

* Please include your Ethics statement in the Methods section of your manuscript.

ABSTRACT

* Please confirm that your abstract complies with our requirements, including format (three sections: Background, Methods and Findings, and Conclusions) and providing all the information relevant to this study type https://journals.plos.org/plosmedicine/s/submission-guidelines#loc-abstract

* Please include the numerical results for the sibling analysis in the abstract.

* In the last sentence of the Abstract Methods and Findings section, please describe the main limitation(s) of the study's methodology.

FUNDING STATEMENT

* The funding statement should include: specific grant numbers, initials of authors who received each award, URLs to sponsors’ websites. Also, please state whether any sponsors or funders (other than the named authors) played any role in study design, data collection and analysis, the decision to publish, or preparation of the manuscript. If they had no role in the research, include this sentence: “The funders had no role in study design, data collection and analysis, decision to publish, or preparation of the manuscript.”

* Please include URLs to the funders’ websites.

DATA AVAILABILITY

* PLOS Medicine requires that the de-identified data underlying the specific results in a published article be made available, without restrictions on access, in a public repository or as Supporting Information at the time of article publication, provided it is legal and ethical to do so. Please see the policy at http://journals.plos.org/plosmedicine/s/data-availability and FAQs at http://journals.plos.org/plosmedicine/s/data-availability#loc-faqs-for-data-policy

* The Data Availability Statement (DAS) requires revision. For each data source used in your study:

a) If the data are freely or publicly available, note this and state the location of the data: within the paper, in Supporting Information files, or in a public repository (include the DOI or accession number).

b) If the data are owned by a third party but freely available upon request, please note this and state the owner of the data set and contact information for data requests (web or email address). Note that a study author cannot be the contact person for the data.

c) If the data are not freely available, please describe briefly the ethical, legal, or contractual restriction that prevents you from sharing it. Please also include an appropriate contact (web or email address) for inquiries (again, this cannot be a study author).

FIGURES AND TABLES

* Please provide titles and legends for all figures and tables (including those in Supporting Information files). Please define all acronyms used in each figure or table in its corresponding legend. Currently the titles are quite long and detailed, and there are no legends. Please amend.

* Please consider if moving some of the figures/tables shared in the supplementary information into the main text would aid the reader. Specifically, we would suggest to move figure S5 to the main manuscript.

OBSERVATIONAL, COHORT, CROSS-SECTIONAL, AND CASE CONTROL STUDIES

* Please ensure that the study is reported according to the STROBE guideline, and include the completed STROBE checklist as Supporting Information. Please add the following statement, or similar, to the Methods: ""This study is reported as per the Strengthening the Reporting of Observational Studies in Epidemiology (STROBE) guideline (S1 Checklist).""

The STROBE guideline can be found here: http://www.equator-network.org/reporting-guidelines/strobe/

When completing the checklist, please use section and paragraph numbers, rather than page numbers.

* Did your study have a prospective protocol or analysis plan? Please state this (either way) early in the Methods section.

a) If a prospective analysis plan (from your funding proposal, IRB or other ethics committee submission, study protocol, or other planning document written before analyzing the data) was used in designing the study, please include the relevant prospectively written document with your revised manuscript as a Supporting Information file to be published alongside your study, and cite it in the Methods section. A legend for this file should be included at the end of your manuscript.

b) If no such document exists, please make sure that the Methods section transparently describes when analyses were planned, and when/why any data-driven changes to analyses took place.

c) In either case, changes in the analysis-- including those made in response to peer review comments-- should be identified as such in the Methods section of the paper, with rationale.

Comments from Reviewers:

Reviewer #1: Thank you very much for addressing my questions. I do not have any further comments.

Reviewer #2: Thank you for responding to my comments. I appreciate, particularly, on showing primary data in new mental health conditions following HPV test positive, which was convincing to the explanation.

I have only one minor suggestion - it isn't immediately clear how 'comorbidity' (as in Figure S1 the DAG) could influence HPV test positive. Could you please expand that in the Methods / Supplementary materials using hyperlipidaemia as an example?

Reviewer #3: Thank you for the revised manuscript and the detailed responses to the reviewers' comments. The authors have addressed most of the concerns raised, and the manuscript has improved considerably. However, one point still requires attention before the manuscript is ready for publication.

In the author response, the authors noted that shared family factors—such as genetic background, early-life experiences, and similar lifestyle habits—may play a stronger role in CVD-related mortality than in CVD incidence, and that this may partly explain why the association was weaker in the sibling-matched analysis. However, this point is not discussed in the revised manuscript. We suggest that the authors include this explanation in the Discussion section.

Reviewer #4: The authors have adequately addressed the comments raised during the previous round of review. They have added analyses of absolute risk differences and population-attributable fractions, incorporated HPV vaccination into the adjusted models, performed additional subgroup and sensitivity analyses, and expanded the Discussion to clarify differences from previous studies. These revisions have strengthened the manuscript and improved its interpretation.

The study is well designed, the analyses are rigorous, and the findings provide important evidence regarding the association between HPV infection and cardiovascular disease. I have no further substantive comments and recommend acceptance of the manuscript in its current form.

Any attachments provided with reviews can be seen via the following link:

[LINK]

# Author comment: Author response to Decision Letter 2

**DOI:** 10.1371/journal.pmed.1005222.r005

24 Jul 2026

Attachment Submitted filename: Response letter_2nd_2.0_260724.docx

# Editor report: Decision Letter 2

**DOI:** 10.1371/journal.pmed.1005222.r006

**Authors:** Suzanne De Bruijn

27 Jul 2026

Dear Dr. Deng,

Thank you very much for re-submitting your manuscript "Human papillomavirus infection and risk of cardiovascular disease: A population-based matched cohort study with sibling analysis" (PMEDICINE-D-26-01654R2) for review by PLOS Medicine. Thank you for addressing our editorial requests. However, we have a few remaining requests, we need you to address before we can accept the paper.

Our remaining requests are:

1) Statistical reporting: Thank you for amending your statistical reporting. However, we note that in the tables you still use round brackets rather than square ones. Please amend, so that the text, the figures and tables all follow our statistical guidelines:

- Please report statistical information as follows to improve clarity for the reader "22% (95% CI [13,28]; p</=)".

- Please separate upper and lower bounds with commas instead of hyphens as the latter can be confused with reporting of negative values.

- Please repeat statistical definitions (HR, CI etc.) for each set of parentheses.

2) Competing interests: Please include a statement “The other authors declare that no competing interests exist.”

3) Data availability: thank you for providing contact details from someone that is not the study author. However, please provide more detail on the requirement for researchers to gain access to the data.

4) Figure 4: Please include the variables that were adjusted for in the legend.

5) This is a mistake on my part, please accept my apologies. In my previous decision, I've asked "Please consider if moving some of the figures/tables shared in the supplementary information into the main text would aid the reader. Specifically, we would suggest to move figure S5 to the main manuscript." However, I did mean Table S5.

a) Please consider whether moving TABLE S5 into the main manuscript would aid the reader.

b) Thank you for moving figure S1 to the main text, due to my erroneous request. I will leave it up to you whether you want to leave this figure in the main text, or move it back to the supplements.

***Please note while forming your response, if your article is accepted, you may have the opportunity to make the peer review history publicly available. The record will include editor decision letters (with reviews) and your responses to reviewer comments. If eligible, we will contact you to opt in or out.***

In revising the manuscript for further consideration here, please ensure you address the specific points made by each reviewer and the editors. In your rebuttal letter you should indicate your response to the reviewers' and editors' comments and the changes you have made in the manuscript. Please submit a clean version of the paper as the main article file. A version with changes marked must also be uploaded as a marked up manuscript file.

In addition to these revisions, you may need to complete some formatting changes, which you will receive in a follow up email. A member of our team will be in touch with a set of requests shortly. If you do not receive a separate email within a few days, please assume that checks have been completed, and no additional changes are required.

We expect to receive your revised manuscript within 1 week. Please email us (plosmedicine@plos.org) if you have any questions or concerns.

Please note, when your manuscript is accepted, an uncorrected proof of your manuscript will be published online ahead of the final version, unless you've already opted out via the online submission form. If, for any reason, you do not want an earlier version of your manuscript published online or are unsure if you have already indicated as such, please let the journal staff know immediately at plosmedicine@plos.org.

If you have any questions in the meantime, please contact me or the journal staff on plosmedicine@plos.org.

We look forward to receiving the revised manuscript by Aug 03 2026 11:59PM.

Sincerely,

Suzanne De Bruijn, PhD

Associate Editor

PLOS Medicine

plosmedicine.org

# Author comment: Author response to Decision Letter 3

**DOI:** 10.1371/journal.pmed.1005222.r007

31 Jul 2026

Attachment Submitted filename: Response letter_3th_1.0_260731.docx

# Editor report: Decision Letter 3

**DOI:** 10.1371/journal.pmed.1005222.r008

**Authors:** Suzanne De Bruijn

3 Aug 2026

Dear Dr Deng,

On behalf of my colleagues and the Academic Editor, Pei Gao, I am pleased to inform you that we have agreed to publish your manuscript "Human papillomavirus infection and risk of cardiovascular disease: A population-based matched cohort study with sibling analysis" (PMEDICINE-D-26-01654R3) in PLOS Medicine.

Before your manuscript can be formally accepted you will need to complete some formatting changes, which you will receive in a follow up email. Please be aware that it may take several days for you to receive this email; during this time no action is required by you. Once you have received these formatting requests, please note that your manuscript will not be scheduled for publication until you have made the required changes.

In the meantime, please log into Editorial Manager at http://www.editorialmanager.com/pmedicine/, click the "Update My Information" link at the top of the page, and update your user information to ensure an efficient production process.

PRESS

We frequently collaborate with press offices. If your institution or institutions have a press office, please notify them about your upcoming paper at this point, to enable them to help maximise its impact. If the press office is planning to promote your findings, we would be grateful if they could coordinate with medicinepress@plos.org. If you have not yet opted out of the early version process, we ask that you notify us immediately of any press plans so that we may do so on your behalf.

We also ask that you take this opportunity to read our Embargo Policy regarding the discussion, promotion and media coverage of work that is yet to be published by PLOS. As your manuscript is not yet published, it is bound by the conditions of our Embargo Policy. Please be aware that this policy is in place both to ensure that any press coverage of your article is fully substantiated and to provide a direct link between such coverage and the published work. For full details of our Embargo Policy, please visit http://www.plos.org/about/media-inquiries/embargo-policy/.

Thank you again for submitting to PLOS Medicine. We look forward to publishing your paper.

Sincerely,

Suzanne De Bruijn, PhD

Associate Editor

PLOS Medicine
